# PTEN interacts with the transcription machinery on chromatin and regulates RNA polymerase II-mediated transcription

**DOI:** 10.1093/nar/gkz272

**Published:** 2019-04-24

**Authors:** Nicole Steinbach, Dan Hasson, Deepti Mathur, Elias E Stratikopoulos, Ravi Sachidanandam, Emily Bernstein, Ramon E Parsons

**Affiliations:** 1 Department of Oncological Sciences, Tisch Cancer Institute, Icahn School of Medicine at Mount Sinai, 1470 Author afMadison Avenue, New York, NY 10029, USA; 2 Graduate School of Biomedical Sciences, Icahn School of Medicine at Mount Sinai, 1470 Madison Avenue, New York, NY 10029, USA

## Abstract

Regulation of RNA polymerase II (RNAPII)-mediated transcription controls cellular phenotypes such as cancer. Phosphatase and tensin homologue deleted on chromosome ten (PTEN), one of the most commonly altered tumor suppressors in cancer, affects transcription via its role in antagonizing the PI3K/AKT signaling pathway. Using co-immunoprecipitations and proximal ligation assays we provide evidence that PTEN interacts with AFF4, RNAPII, CDK9, cyclin T1, XPB and CDK7. Using ChIP-seq, we show that PTEN co-localizes with RNAPII and binds to chromatin in promoter and putative enhancer regions identified by histone modifications. Furthermore, we show that loss of PTEN affects RNAPII occupancy in gene bodies and further correlates with gene expression changes. Interestingly, PTEN binds to promoters and negatively regulates the expression of genes involved in transcription including *AFF4* and *POL2RA*, which encodes a subunit of RNAPII. Loss of PTEN also increased cells’ sensitivity to transcription inhibition via small molecules, which could provide a strategy to target PTEN-deficient cancers. Overall, our work describes a previously unappreciated role of nuclear PTEN, which by interacting with the transcription machinery in the context of chromatin exerts an additional layer of regulatory control on RNAPII-mediated transcription.

## INTRODUCTION

Phosphatase and tensin homologue deleted on chromosome ten (PTEN) is a dual specificity phosphatase that is inactivated or lost in a large proportion of human cancers, making it one of the most commonly mutated tumor suppressors (1,2). PTEN is best known for its function as a lipid phosphatase at the plasma membrane, where it dephosphorylates phosphatidylinositol-(3,4,5)-trisphosphate (PIP3), thereby antagonizing the phosphoinositide 3-kinase (PI3K) signaling pathway (3–5). In addition to its essential role at the membrane, PTEN has been found localized to the nucleus of non-malignant and malignant tissues (6). The loss of nuclear PTEN has been associated with more aggressive tumors and the malignant transformation of normal cells (6). Although lipid phosphoinositides and other components of the PI3K/AKT pathway have been found in the nucleus, only the cytoplasmic pools of PIP3 are sensitive to PTEN phosphatase activity (7). Consistent with this, functions of nuclear PTEN, including homologous recombination-mediated repair of double-strand DNA breaks (8,9), cell proliferation, and chromatin condensation (10,11), are mostly independent of its lipid phosphatase activity.

It has been described that loss of PTEN alters gene expression programs associated with poor prognosis (12–14). PTEN has been shown to repress RNA polymerase I (RNAPI)- (15) and RNA polymerase III (RNAPIII)-mediated transcription (16), dependent on PTEN’s inhibitory role in the PI3K/AKT/mTOR/S6K signaling pathway. However, little is known about PTEN’s direct role in RNA polymerase II (RNAPII)-mediated transcription.

RNAPII-mediated transcription is a tightly regulated process, which is coupled to phosphorylation events on the highly conserved heptad repeat (Y_1_S_2_P_3_T_4_S_5_P_6_S_7_) of the RNAPII subunit RPB1 C-terminal domain (CTD) (17). During initiation, RNAPII Serine 5 and Serine 7 get phosphorylated (RNAPII Ser5P and Ser7P) by the cyclin-dependent kinase 7 (CDK7) (18), which together with cyclin H and Mat1 comprises a trimeric complex that can associate with the basal transcription factor IIH (TFIIH) ten subunit core complex (19). Upon entering productive elongation levels of RNAPII Ser5P drop and levels of RNAPII Ser2P increase. Phosphorylation of Ser2 is thought to be dependent on the positive elongation factor (P-TEFb) comprising the cyclin-dependent kinase 9 (CDK9) as a catalytic subunit, and cyclin T1 or cyclin T2 as a regulatory subunit (20,21), as well as on the cyclin-dependent kinases 12 and 13 (CDK12 and 13) in complex with cyclin K (22). P-TEFb can be part of several multi-subunit complexes including the super elongation complex (SEC) (23). The scaffold for assembling the SEC is AF4/FMR2 family member 4 (AFF4), which belongs to the mammalian AFF family (24). The P-TEFb complex is also involved in overcoming promoter-proximal pausing of RNAPII by phosphorylating negative elongation factor (NELF) and transcription elongation factor SPT5 (25). The activity of the P-TEFb complex can be positively influenced, for example, by CDK7 (26) and the bromodomain protein BRD4 (27) and negatively by association with the inhibitory 7SK small nuclear ribonulceoprotein (7SK snRNP) complex comprising the 7SK snRNA and hexamethylene bisacetamide-inducible proteins 1 and 2 (HEXIM1 and HEXIM2) (25).

As transcription is an essential process in normal as well as malignant cells and tissues, it has long been regarded a challenging target in cancer therapy. RNAPII-mediated transcription can be inhibited at different stages during the transcription cycle, for example during initiation or elongation. CDK7 kinase activity can be effectively inhibited by the covalent CDK7 inhibitor THZ1 (28). Transcription initiation can also be blocked by Triptolide, a drug that targets the XPB subunit of TFIIH and inhibits its DNA-dependent ATPase activity (29). Elongation can be inhibited by targeting CDK9 activity by Flavopiridol (30) or LCD000067 (31).

Here, we report that nuclear PTEN binds to components of the transcription machinery and that its genome-wide distribution in chromatin correlates with RNAPII, H3K4me3, and H3K27ac at promoters, and H3K27ac and H3K4me1 in distal regions. Loss of PTEN increases levels of RNAPII Ser2P and Ser5P that in turn leads to a genome-wide increase in RNAPII Ser2P and Ser5P occupancy in gene bodies and altered gene expression. Changes in chromatin occupancy and gene expression due to PTEN mutation correlated most strongly at PTEN-bound genes, which included components of the transcription machinery and transcriptional regulators. PTEN loss also upregulates a subset of genes, whose expression renders cells more susceptible to transcription inhibition by small molecule inhibitors. In summary, our findings indicate that PTEN functions in the nucleus, where by interacting with the transcription machinery it provides an additional layer of regulatory control on RNAPII-mediated transcription and gene expression.

## MATERIALS AND METHODS

### Plasmids and constructs

Bacterial expression plasmids encoding GST-PTEN domains have been reported previously (32). The MSCVneo-*PTEN* vector and the pIRES-FLAG-C2TAIL vector were previously described (33). The PTEN phosphatase-domain point mutants were generated using the QuikChange II XL Site-Directed mutagenesis kit (Agilent) as per manufacturer's instructions utilizing the MSCVneo-*PTEN* as a template. The AFF4 bait-fragment was cloned into the pcDNA^TM^3.1/V5-His vector using the TOPO^®^ TA Expression Kit (Invitrogen) via topoisomerase ligation of Taq-Polymerase generated PCR purified fragments using full length AFF4 as a template.

### Antibodies

All antibodies are listed in the Supplementary Materials and Methods.

### Cell lines

MycOE Pik3ca Mut cells, MycOE Pten Null cells and mouse embryonic fibroblasts were cultured in Dulbecco's minimal essential medium (Cellgro) supplemented with 10% (vol/vol) fetal bovine serum (FBS), 100 IU penicillin, 100 μg/ml streptomycin (Cellgro) and 2 mM l-glutamine (total 6 mM l-glutamine) (Cellgro). HEK293 and HeLa cells were cultured in Dulbecco's minimal essential medium (Cellgro) supplemented with 10% (vol/vol) fetal bovine serum (FBS), 100 IU penicillin, 100 μg/ml streptomycin (Cellgro). DBTRG-05MG and HCT116 cells were cultured in RPMI-1640 supplemented with 10% (vol/vol) FBS, 100 IU penicillin and 100 μg/ml streptomycin.

### Generation of primary Mouse Embryonic Fibroblasts (MEFs)

*Pten* ^flox/flox^ mice (34) in a C57bl/6 background were interbred. Embryos were harvested at E14.5 and cultured in DMEM (Cellgro) supplemented with 10% (vol/vol) fetal bovine serum (FBS), 100 IU penicillin, 100 μg/ml streptomycin (Cellgro) and 2 mM l-glutamine (total 6 mM l-glutamine) (Cellgro). For deletion of Pten, MEFs were infected with adenovirus Adeno-CMV-Cre-recombinase (Vector Biolabs) or Adeno-CMV-Null (Vector Biolabs) as a control.

### CRISPR clone generation

The pL-CRISPR.EFS.GFP vector containing the *PTEN* targeting guide RNA (ACAGATTGTATATCTTGTAA NGG) was generated previously (35) (Addgene, plasmid 57818). Lentivirus was produced in HEK-293T cells as previously described (36). GFP-positive HeLa cells were isolated using fluorescence activated cell sorting, and clonal cell populations were assessed for PTEN protein levels by immunoblotting. DNA from single colonies was amplified and sequenced by Genewiz using the primers listed in the Supplementary Materials and Methods.

### Generation of PTEN overexpressing cell lines

Retrovirus was produced by transfecting MSCVneo-*PTEN* WT and point mutation constructs into Phoenix packaging cells as previously described (37). MycOE Pten Null cells were infected, selected using G418 selection antibiotic, and assessed for PTEN protein levels by immunoblotting.

### Cell proliferation assays

1500 cells were plated per well in a 96-well plate in triplicate for each experiment. Proliferation was monitored by analyzing the occupied area (% confluence) of cell images over time as recorded by the IncuCyte ZOOM^®^ live-cell imaging and analysis system (Essen BioScience).

### Cell fractionation

Cell fractionation was performed as described before (38). The detailed protocol can be found in the Supplementary Materials and Methods.

### Co-immunoprecipitation of V5- or FLAG-tagged proteins

HEK293 cells were co-transfected with FLAG-tagged PTEN-C2TAIL and V5-tagged AFF4 or empty vector (EV), respectively. Cells were lysed in BC200 buffer (25 mM Tris pH 7.5, 200 mM NaCl, 1 mM EDTA, 0.2% Triton X-100, 0.2% glycerol), sonicated, centrifuged and pre-cleared with protein A/G agarose beads. Supernatants were incubated with anti-V5 agarose beads, beads were washed with TBS (10 mM Tris pH 7.4, 150 mM NaCl, 15 mM MgCl_2_) and proteins were eluted using 0.5 mg/ml V5 peptide (Sigma-Aldrich).

### GST fusion protein purification and GST bead pull downs

GST fusion proteins were purified as previously described (32). *In vitro* transcribed/translated AFF4, pre-cleared supernatants of HEK293 or DBTRG cells were incubated with GST-PTEN or indicated GST-PTEN domains loaded onto glutathione sepharose beads. Beads were washed and proteins were eluted with elution buffer (25 mM Tris pH 8.0, 150 mM NaCl, 50 mM glutathione). The extended protocol is provided in the Supplementary Materials and Methods.

### Endogenous co-immunoprecipitations

HEK293 cells were lysed in BC200 (25 mM Tris pH 7.5, 200 mM NaCl, 1 mM EDTA, 0.2% Triton X-100, 0.2% Glycerol). Lysates were sonicated, centrifuged, and then pre-cleared using Pierce™ protein A/G magnetic beads (ThermoFisher Scientific). Supernatants were incubated with PTEN (6H2.1) or mouse IgG crosslinked to Pierce™ protein A/G magnetic beads using dimethyl adipimidate (ThermoFisher Scientific). Beads were washed four times with BC200 and proteins were eluted with 0.1 M glycine pH 2.0.

### Proximal ligation assay (PLA)

PLA assays were performed according to the manufacturer's protocol (DuoLink^®^ In Situ Red Starter kit mouse/rabbit or goat/rabbit, Sigma-Aldrich). In brief, 20 000 HeLa cells were plated on gelatin-coated, 16-well chamber slides, fixed with 2% Paraformaldehyde, permeablized in perm/block solution (10% donkey serum, 0.1% Triton X-100 in PBS) and incubated with primary antibodies diluted 1:100 overnight at 4°C. PLA detection was performed and images were taken with a confocal microscope (Zeiss LSM 880 with Airyscan) at a magnification of 63x and analyzed with the ImageJ software.

### mRNA-Seq

1 μg of total RNA per sample from three independent biological replicates of PTEN WT and CRISPR-*PTEN* HeLa cells was processed using the TruSeq RNA Sample Preparation kit V2 (Illumina). Libraries were sequenced using the NextSeq^®^ 500/550 High output Kit v2 (75 cycles) on a NextSeq^®^ 500 sequencing system (Illumina). mRNA-seq analysis was performed as previously described (39).

### ChIP experiments, library preparation, sequencing and data analysis

One-step and two-step crosslinking ChIP experiments were performed as described before (40,41). One-step crosslinking was performed using 1% formaldehyde for 10 min at RT and two-step crosslinking was performed using 2 mM disuccinimidyl glutarate (Sigma-Aldrich) for 45 min at RT, followed by 1% formaldehyde for 10 min at RT. Chromatin was sheared with a UCD-400 Bioruptor (Diagenode) for 12–40 cycles (30 s on/30 s off). 2–10 ng of DNA were used to generate ChIP-seq libraries as described before (42) and samples were submitted to 75 bp single-end sequencing using the NextSeq^®^ 500/550 High output Kit v2 (75 cycles) on a NextSeq^®^ 500 sequencing system (Illumina). Extended protocols and the data analysis procedure are provided in the Supplementary Materials and Methods.

## RESULTS

### Nuclear PTEN associates with chromatin

**Figure 1.**
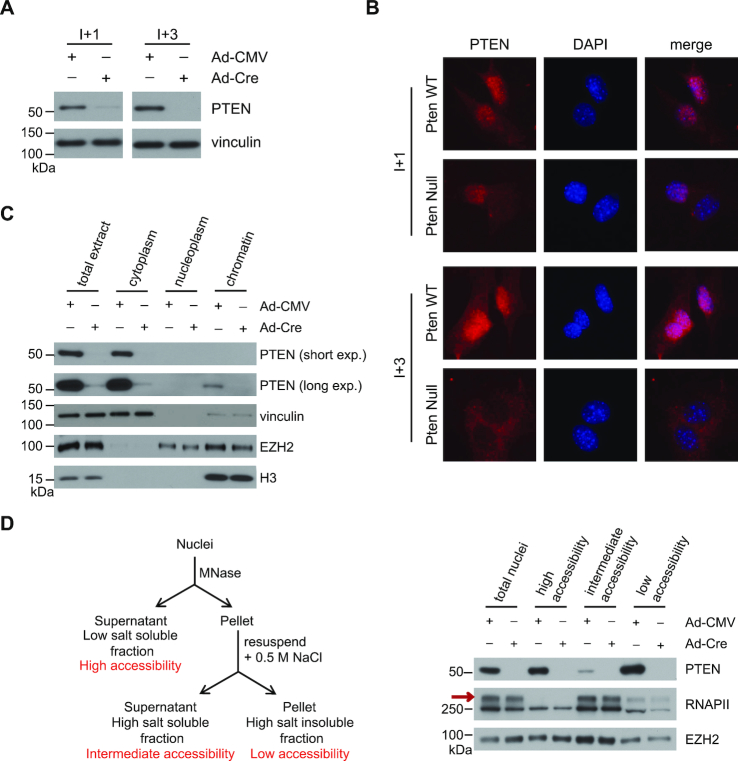
Nuclear Pten associates with chromatin. (**A**) Immunoblot analysis of whole cell lysates of Pten WT and Pten^−/–^ MEFs for PTEN expression. Lysates were taken 1 (I+1) or 3 (I+3) passages after infection with Adeno-Cre containing virus. (**B**) Representative immunofluorescence images of Pten WT and Pten^−/–^ MEFs stained for PTEN and DAPI. Cells were fixed 1 (I+1) or 3 (I+3) passages after infection with Adeno-Cre containing virus. (**C**) Subcellular fractionation of Pten WT and Pten^−/–^ MEFs. PTEN, vinculin, EZH2, and H3 protein levels were determined by immunoblotting. (**D**) (Left) Schematic representation of the MNase/high-salt fractionation protocol. (Right) Partial MNase digestion followed by high-salt extraction was performed on nuclei extracted from Pten WT and Pten^−/−^ MEFs. PTEN, RNAPII, and EZH2 protein levels were determined by immunoblotting. RNAPII0 is indicated by a red arrow.

Nuclear PTEN has been extensively studied in mouse embryonic fibroblasts (MEFs), where it was shown to be associated with chromatin (9–11). To further understand the roles of nuclear PTEN and its function in chromatin regulation, we used primary MEFs that have loxP sites flanking exon 5 inserted into the endogenous locus of *Pten* (34). By immunoblot, as early as one passage post infection with Adeno-Cre-containing virus, almost 100% of Pten is lost and is virtually undetectable three passages after infection (Figure 1A). However, immunofluorescence (IF) revealed that one passage post infection only cytosolic Pten is lost while nuclear Pten remained visible in >50% of the cells (Figure 1B). Nuclear Pten is undetectable three passages post infection (Figure 1B), suggesting that the nuclear pool of Pten might be distinct from the cytosolic pool.

To further characterize the Pten nuclear pool, we performed cellular fractionation experiments. As expected the majority of Pten resided in the cytosolic fraction (Figure 1C). However, under high-salt conditions Pten is also found at relatively lower levels within the insoluble nuclear fraction that contains the nuclear lamina and chromatin, but not in the soluble nucleoplasm (Figure 1C). We next performed partial micrococcal nuclease (MNase) digestion followed by high-salt extraction, to differentiate between various levels of euchromatin accessibility (i.e. high, intermediate, and low) (Figure 1D; left). A large fraction of Pten associated with the soluble nuclear proteins, and with the least accessible, higher order euchromatin (Figure 1D; right). However, a small fraction of Pten is associated with chromatin with intermediate accessibility (Figure 1D; right). This fraction also displayed high levels of RNAPII0, the highly phosphorylated form of RNAPII that is associated with transcriptional activity (Figure 1D; right, red arrow) (17). These results suggest that nuclear PTEN can associate with transcriptionally active chromatin.

### Nuclear PTEN interacts with components of the transcription machinery

**Figure 2.**
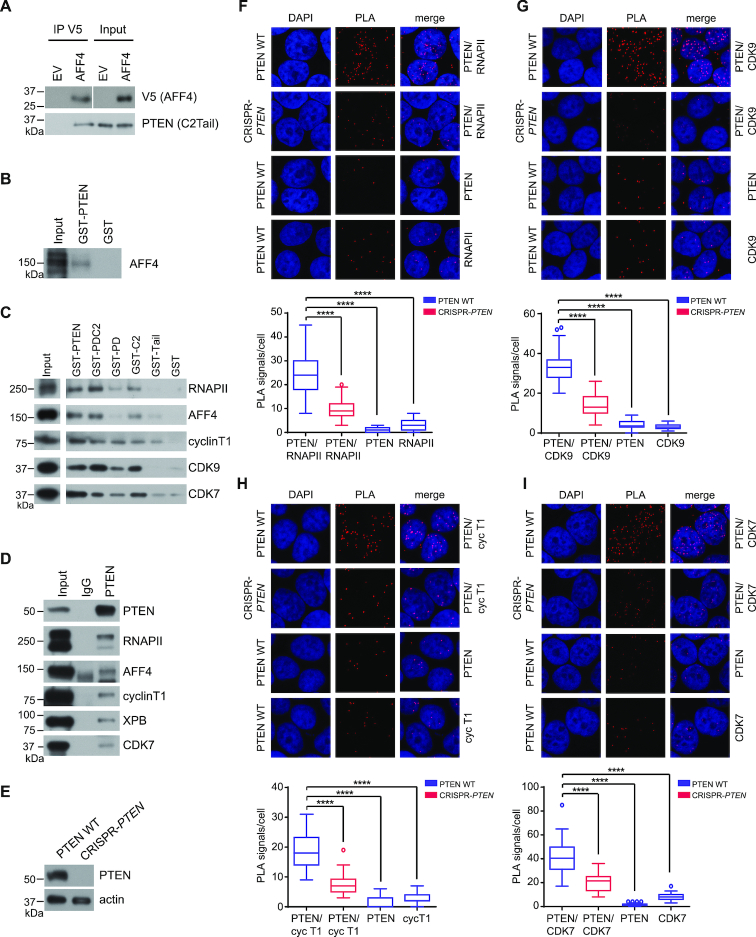
Recombinant and endogenous PTEN can bind to proteins of the transcription machinery. (**A**) HEK293 cell lysates co-expressing the V5-tagged prey-fragment (AFF4) and the FLAG-tagged bait-fragment (PTEN) were incubated with anti-V5 agarose beads. V5 (AFF4) and PTEN (C2TAIL) protein levels were determined by immunoblotting. (**B**) *In vitro* translated/transcribed full-length AFF4 was incubated with GST-PTEN or GST sepharose beads. AFF4 protein levels were determined by immunoblotting. (**C**) HEK293 cell lysates were incubated with GST-PTEN, GST-PDC2, GST-PD, GST-C2, GST-TAIL or GST sepharose beads. Pulled-down protein complexes were analyzed for RNAPII, AFF4, cyclin T1, CDK9 and CDK7 protein levels by immunoblotting. (**D**) HEK293 cell lysates were incubated with PTEN (6H2.1) or mouse IgG. Pulled-down protein complexes were analyzed for PTEN, RNAPII, AFF4, cyclin T1, XPB and CDK7 protein levels by immunoblotting. (**E**) Immunoblot analysis of whole cell lysates of PTEN WT and CRISPR-*PTEN* HeLa cells for PTEN expression. (F–I) Representative images of proximal ligation assays performed in PTEN WT and CRISPR-*PTEN* HeLa cells. For each condition the PTEN antibody (Cat #9559, Cell Signaling) was incubated with a (**F**) RNAPII, (**G**) CDK9, (**H**) cyclin T1 or (**I**) CDK7 antibody. As a control each antibody was incubated individually. The quantification for each condition is shown below each panel. Tukey boxplots, *n* = 40, Student's *t*-test, ****P ≤ 0.0001.

To reveal novel functions of nuclear PTEN, we performed a yeast-2-hybrid (Y2H) screen using the PTEN C2TAIL-domain, which is thought to facilitate most of PTEN’s known interactions with other proteins (43), as the bait-fragment against a library of prey-fragments of the human fetal brain. The screen identified AFF4, the scaffolding protein of the SEC (23), as a potential interaction partner of PTEN (Supplementary Table S1). This interaction was confirmed by co-immunoprecipitation experiments from HEK293 cell lysates expressing the PTEN C2TAIL and V5-tagged AFF4 fragments (Figure 2A and Supplementary Figure S1A) and by pull-down experiments using full-length GST-PTEN or the phosphatase-dead mutant GST-PTEN C124S purified from bacteria and *in vitro* transcribed/translated full-length AFF4 (Figure 2B, Supplementary Figure S1B and C). GST-PTEN also interacted with endogenous AFF4 from PTEN-deficient DBTRG-05MG glioblastoma (33) and HEK293 cell lysates (Supplementary Figure S1D), indicating that PTEN can bind to a component of the transcription machinery. Moreover, GST-PTEN and GST-PTEN fragments containing the phosphatase (PD) and C2 domain interacted with AFF4 interactors and P-TEFb components cyclin T1 and CDK9 (Figure 2C, Supplementary Figure S1E and S1F) (23). PTEN and the same fragments also interacted with RNAPII, which binds to P-TEFb, and to CDK7, which phosphorylates the RNAPII CTD (Figure 2C) (17), indicating that PTEN can interact with additional components of the transcription machinery. The PTEN Tail-region seems dispensable for these interactions as several GST-PTEN deletion constructs, which are missing the tail still bind to these proteins, whereas the Tail-domain by itself only weakly interacts with cyclin T1 and CDK7 and very weakly interacts with AFF4 (Figure 2C). Further, the phosphatase activity of PTEN was not required for interactions of PTEN with the transcription machinery, as both GST-PTEN and the phosphatase-dead mutant GST-PTEN C124S interacted with AFF4, SPT5, HEXIM1, CDK9, XPB and CDK7 (Supplementary Figure S1G).

In addition, immunoprecipitations revealed endogenous interactions between PTEN and AFF4, as well as RNAPII, cyclin T1, XPB and CDK7 (Figure 2D). The endogenous interactions were confirmed using proximity ligation assays (PLA), in which positive interactions are represented by distinct fluorescent spots. We created clonal HeLa cell lines in which *PTEN* was deleted employing the CRISPR/Cas9 system using a guide RNA targeting exon 3 and a small part of the downstream intron of the endogenous *PTEN* locus (CRISPR-*PTEN*) (Figure 2E and Supplementary Figure S2) (35). We find significantly more fluorescent spots in PTEN WT compared to CRISPR-*PTEN* HeLa cells, when using two antibodies directed against PTEN and RNAPII, cyclin T1, CDK9, or CDK7 (Figure 2F–I). Using either antibody alone resulted in unspecific background signals, as expected (Figure 2F–I). Collectively, these data suggest that PTEN can bind to components of the transcription machinery in the nucleus.

Moreover, we analyzed nuclear extracts from PTEN WT HeLa cells by size exclusion chromatography, which is a useful tool to isolate large multi-subunit complexes (i.e. transcription complexes) and to identify potential interactors (23). These studies indicate that while most PTEN elutes around 75 kDa corresponding to unbound PTEN, a small fraction of PTEN, elutes in fractions corresponding to 669 and 2 MDa (Supplementary Figure S3). We further found components of the transcription machinery such as RNAPII, RNAPII Ser2P and Ser5P, BRD4, SPT5, and HEXIM1 as well as components of the TFIIH complex (CDK7 and cyclin H) and the SEC (AFF4 and P-TEFb components CDK9 and cyclin T1), to elute within this size range (Supplementary Figure S3) suggesting that nuclear PTEN migrates with large multi-subunit complexes containing components of the transcription machinery.

### PTEN binds to chromatin in promoter regions and affects expression of a subset of genes

**Figure 3.**
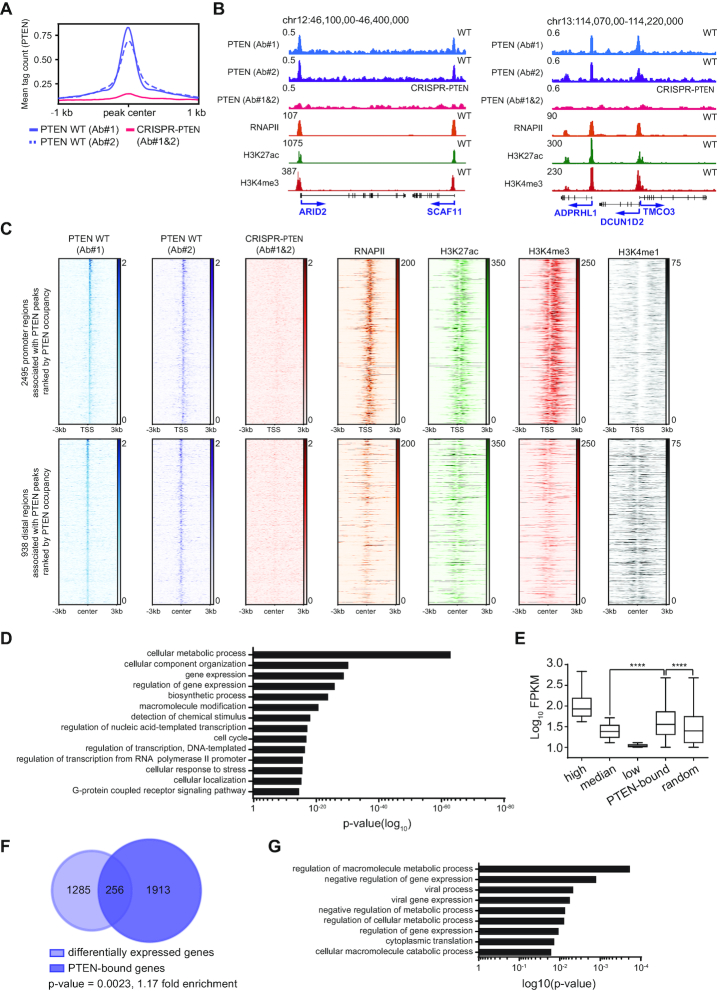
PTEN binds to chromatin in promoter and putative enhancer regions. (**A**) PTEN ChIP-seq meta-profiles using two PTEN antibodies in PTEN WT (blue) and CRISPR-*PTEN* (red) HeLa cells. Data are centered and ±1 kb are shown. Plots represent average read counts per 50 bp bins. (**B**) Representative captures of the UCSC genome browser (GRCh37/hg19) showing PTEN, and publicly available RNAPII (44,45), H3K27ac, H3K4me1 and H3K4me3 (46,47) tracks in HeLa cells. The y-axis represents normalized read counts in reads per million. (**C**) Heatmaps of ChIP-seq reads for PTEN, and publicly available RNAPII (44,45), H3K27ac, H3K4me3 and H3K4me1 (46,47). Data are centered and ±3 kb are shown for (top) 2495 promoter regions and (bottom) 938 distal regions rank-ordered by PTEN occupancy. (**D**) GO enrichment analysis of PTEN-bound genes. p-values are given. (**E**) Log_10_FPKM of 3830 high, 7660 median, and 3830 low expressed genes, 2672 PTEN-bound and 2672 random genes in PTEN WT HeLa cells. Tukey boxplots with outliers omitted. Student's *t*-test, ****P ≤ 0.0001. (**F**) Hypergeometric gene set enrichment analysis to determine the overlap between differentially expressed and PTEN-bound genes. *P*-value and fold-enrichment are given. Calculation was based on 15 330 genes. (**G**) GO enrichment analysis of PTEN-bound genes that were significantly downregulated after PTEN loss. p-values are given.

To further explore the relationship between chromatin-bound PTEN and the transcription machinery genome-wide, we conducted chromatin immunoprecipitation coupled to high-throughput sequencing (ChIP-seq) in PTEN WT and CRISPR-*PTEN* HeLa cells using two different antibodies against PTEN (Figure 3A) (41). By overlapping the peaks yielded by the two PTEN antibodies, we identified 3,433 significant peaks (significance cut-off of –log_10_(*q*-value) > 5), in the PTEN WT cells (Figure 3A; blue, B and C) and PTEN deletion led to a complete loss of these peaks (Figure 3A; red, B and C). 73% of PTEN peaks were located within 1 kb of a transcription start site (TSS) and coincided with RNAPII (44,45), H3K4me3 and H3K27ac, but not H3K4me1 (46,47) (Figure 3B and C; top). PTEN peaks in distal regions coincided predominantly with H3K27ac and H3K4me1, which are markers of active enhancers (48) (Figure 3C; bottom). HOMER *de novo* motif analysis revealed that PTEN peaks in promoter regions coincide preferentially with GC-rich motifs, and this preference was less pronounced in distal regions (Supplementary Figure S4A). As TSSs are often GC-rich, enriched for RNAPII, H3K27ac and H3K4me3, but not H3K4me1 (49) this is consistent with the majority of PTEN being bound to promoters.

To examine the functions of all PTEN-bound genes, we performed gene ontology (GO) analysis. This identified gene sets involved in important cellular processes including cellular metabolism, cell cycle, as well as gene expression (50,51) (Figure 3D), suggesting that PTEN can bind to promoters or distal regions of genes that have functions in key cellular processes including the regulation of transcription.

We next wanted to examine PTEN-bound genes and their gene expression levels and generated RNA-sequencing (RNA-seq) data from PTEN WT and CRISPR-*PTEN* HeLa cell lines. Notably, PTEN-bound genes are expressed at higher than median levels (Figure 3E) and loss of PTEN affects the expression levels of a significant number of these genes in both the up and down direction in roughly equal proportions (Figure 3F and Supplementary Figure S4B). Genes with PTEN bound at the promoter, which were significantly upregulated after PTEN loss included a number of transcription machinery components, most notably, AFF4, POLR2A, which encodes the RPB1 subunit of RNAPII, HEXIM1, MED22 and BRD2 (Supplementary Figure S4B, left). Moreover, GO analysis of genes with PTEN bound at the promoters, which were significantly downregulated after PTEN loss revealed enrichment of gene sets, including ‘regulation of gene expression’ and ‘negative regulation of gene expression’ (Figure 3G and Supplementary Figure S4B; right), further suggesting that PTEN plays a role in transcription regulation.

### PTEN loss increases RNAPII Ser2 and Ser5 phosphorylation and genome-wide occupancy in gene bodies, and alters gene expression

**Figure 4.**
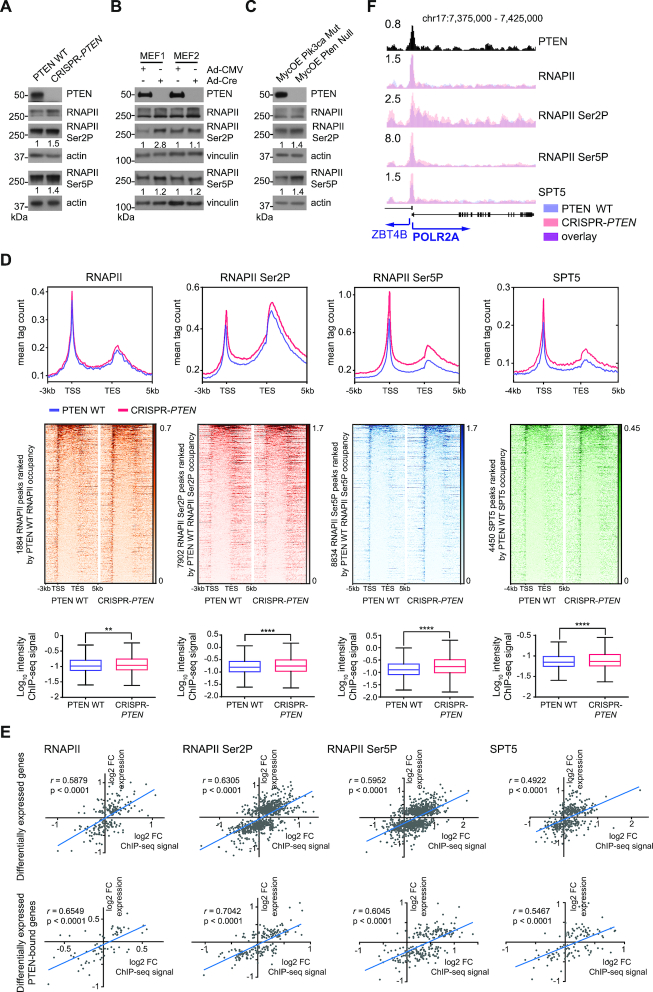
PTEN loss affects RNAPII, RNAPII Ser2P and Ser5P and SPT5 occupancy in chromatin and correlates with gene expression. (A-C) Immunoblot analysis of whole cell lysates from (**A**) PTEN WT and CRISPR-*PTEN* HeLa cells, (**B**) Pten WT and Pten^−/-^ MEFs and (**C**) MycOE Pik3ca Mut and MycOE Pten Null mouse breast cells. The ratio of the RNAPII Ser2P and RNAPII Ser5P bands over the loading control by densitometry was calculated for each sample and normalized to the PTEN WT sample. (**D**) Analysis of RNAPII, RNAPII Ser2P, RNAPII Ser5P and SPT5 ChIP-seq experiments in PTEN WT (blue) and CRISPR-*PTEN* (red) HeLa cells. (Top) ChIP-Seq meta-profiles. Gene bodies were scaled to 5 kb. For RNAPII and RNAPII Ser2P 3 kb up- and 5 kb downstream, for RNAPII Ser5P 5 kb up- and 5 kb downstream, and for SPT5 4 kb up- and 5 kb downstream of gene bodies are shown. (Middle) Heatmaps for ChIP-seq reads. Plots represent average read counts per 50 bp bins. Data is rank-ordered according to occupancy in PTEN WT cells. (Bottom) Log_10_intensity of ChIP-seq signal in genes. Tukey boxplots with outliers omitted. Student's *t*-test, ****P ≤ 0.0001, **P ≤ 0.01. (**E**) (Top and bottom) Pearson correlation of log_2_FC FPKM (FC, fold-change; FPKM, fragments per kilobase of transcript per million mapped reads) and log_2_FC ChIP-Seq signals for RNAPII, RNAPII Ser2P and Ser5P and SPT5 at (top) differentially expressed or (bottom) differentially expressed PTEN-bound genes in PTEN WT and CRISPR-*PTEN* HeLa cells. Pearson correlation coefficients and p-values are given. (F) Capture of the UCSC genome browser (GRCh37/hg19) showing PTEN, RNAPII, RNAPII Ser2P, RNAPII Ser5P and SPT5 at POLR2A in PTEN WT (blue) and CRSIPR-*PTEN* (red) HeLa cells. The y-axis represents normalized read counts in reads per million.

Transcription regulation is highly complex and can be accomplished through a multitude of mechanisms including the phosphorylation of the RNAPII CTD (17). Inspection of whole cell lysates revealed increased levels of RNAPII Ser2P and Ser5P in CRISPR-*PTEN* HeLa cells, as well as in Pten^−/−^ MEFs and a Pten^−/−^ mouse breast tumor cell line (MycOE Pten Null) (52) (Figure 4A–C) when compared to PTEN WT HeLa cells.

Elevated levels of RNAPII Ser2P and Ser5P could indicate an increase in chromatin-bound RNAPII. To test this hypothesis, we conducted ChIP-seq for total RNAPII, RNAPII Ser2P and Ser5P, and SPT5 in PTEN WT and CRISPR-*PTEN* HeLa cells (41). As expected, we found these proteins enriched at promoters, and to a lesser extent at gene bodies in PTEN WT cells (Figure 4D). Upon loss of PTEN, we observed a significant increase in their average enrichment (Figure 4D), suggesting that PTEN loss affects levels of RNAPII in chromatin. In addition, genome-wide changes in chromatin occupancy of RNAPII, RNAPII Ser2P and Ser5P and SPT5 correlated with gene expression changes due to PTEN loss (Supplementary Figure S5A). An increase of RNAPII on TSS could stem from a decrease in the promoter clearance rate (PRR). However, the PRR was not significantly different in PTEN WT and CRISPR-*PTEN* HeLa cells (Supplementary Figure S5B), indicating that PTEN loss does not affect the promoter clearance of RNAPII.

Further RNAPII ChIP-seq experiments revealed significantly higher RNAPII levels at TSS in Pten^−/−^ versus Pten WT MEFs (Supplementary Figure S6A), and at TSS and throughout the entire gene body in MycOE Pten Null versus MycOE Pik3caMut mouse breast tumor cells (Supplementary Figure S6B) (40). RNA microarray expression data from isogenic pairs of primary Pten WT and Pten^−/−^ MEFs was available and showed that PTEN loss induced changes in RNAPII occupancy were correlated with gene expression changes (Supplementary Figure S6C). Overall, these data suggest that in 3 different cell line systems, PTEN loss increases RNAPII occupancy in chromatin, which in turn affects gene expression.

### Levels of RNAPII in chromatin and gene expression correlate strongly at differentially expressed PTEN-bound genes

We next wanted to examine the changes in chromatin occupancy of RNAPII, RNAPII Ser2P and Ser5P, and SPT5 at PTEN-bound genes. The correlation between chromatin occupancy and gene expression was slightly stronger at PTEN-bound genes than at genes genome-wide (Supplementary Figure S5A and C), indicating that expression levels of PTEN-bound genes are sensitive to PTEN loss.

As changes in chromatin occupancy are not always reflected in gene expression changes, we analyzed these relationships at differentially expressed and differentially expressed PTEN-bound genes. As expected, the correlations of changes in chromatin occupancy and gene expression were stronger at differentially expressed genes than at genes genome-wide (Figure 4E; top). Moreover, differentially expressed PTEN-bound genes displayed the strongest correlations between changes in chromatin occupancy and gene expression (Figure 4E; bottom). Notably, this group includes components of the transcription machinery such as POLR2A (Figure 4F and Supplementary Figure S4B), the CTD-containing RPB1 subunit of RNAPII (17). These data suggest that PTEN can bind to promoters of transcription machinery components to regulate their expression, which in turn could affect transcription and gene expression globally.

We next over-expressed wild type PTEN (PTEN WT) in our PTEN-depleted CRISPR-*PTEN* HeLa cells and performed RT-qPCR to probe for changes in gene expression before and after PTEN re-expression. As expected loss of PTEN did increase the expression levels of several of the PTEN-bound genes examined (Supplementary Figure S7). Re-expression of PTEN WT in CRISPR-*PTEN* HeLa cells decreased the expression of three out of four genes tested (Supplementary Figure S7), indicating that PTEN WT can rescue the transcription activation seen in a subset of genes after loss of PTEN.

### PTEN loss sensitizes cells to transcriptional inhibition

**Figure 5.**
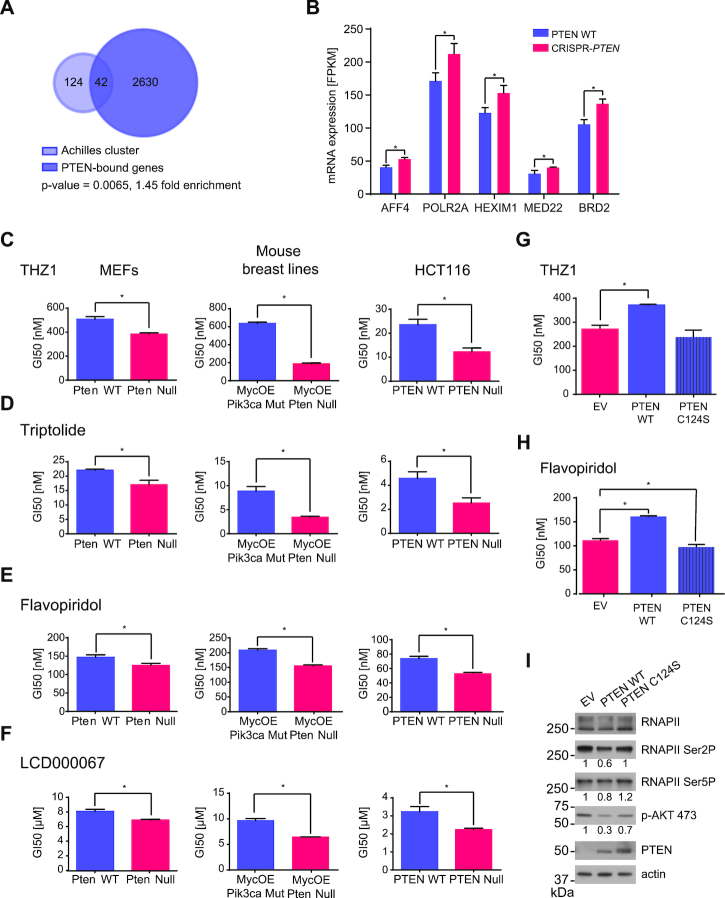
PTEN loss confers sensitivity to transcription inhibition. (**A**) Hypergeometric gene set enrichment analysis of the ‘Achilles cluster’ gene set (53) and PTEN-bound genes. *P*-value and fold-enrichment are given. Calculation was based on 15 330 genes. (**B**) mRNA expression levels as determined by mRNA-seq of AFF4, POLR2A, HEXIM1, MED22 and BRD2 in PTEN WT and CRISPR-*PTEN* HeLa cells. Data are presented as mean ± SD (*n* = 3). Student's *t*-test, *P ≤ 0.05. (C–F) GI50 values of Pten WT and Pten^−/−^ MEFs, MycOE Pik3ca Mut and MycOE Pten Null mouse breast lines and PTEN WT and PTEN^−/−^ HCT116 cells treated with dose titrations of (**C**) THZ1, (**D**) Triptolide, (**E**) Flavopiridol or (**F**) LCD000067. Data are presented as mean ± SD (*n* = 3). Student's *t*-test, *P ≤ 0.05. (G and H) GI50 values of MycOE Pten Null mouse breast tumor lines expressing EV control, PTEN WT or PTEN C124S treated with dose titrations of (**G**) THZ1 or (**H**) Flavopiridol. Data are presented as mean ± SD (*n* = 3). Student's *t*-test, *P ≤ 0.05. (**I**) Whole cell lysates of MycOE Pten Null mouse breast tumor lines expressing EV control, PTEN WT or PTEN C124S were analyzed for protein levels of PTEN, RNAPII, RNAPII Ser2P and Ser5P, and p-AKT 473 by immunoblotting. The ratio of the RNAPII Ser2P and Ser5P, or p-AKT 473 bands over the loading control by densitometry was calculated for each sample and normalized to the MycOE Pten Null EV control sample.

Overexpression of genes involved in cell signaling and transcription has been to shown to render cells more susceptible to transcription inhibition with small molecule inhibitors such as the CDK7 inhibitor THZ1 (28,53). Notably, the ‘Achilles cluster’ a gene set identified in triple negative breast cancer (TNBC), which is thought to confer sensitivity to treatment with THZ1 was significantly over-represented within PTEN-bound genes (Figure 5A) (28,53). This, combined with PTEN’s interaction with the transcription machinery and the PTEN loss-induced upregulation of the transcriptional regulators AFF4, POLR2A, HEXIM1, MED22 and BRD2 (Figure 5B) led us to investigate whether total PTEN loss could render cells more susceptible to transcription inhibition.

We tested the transcription inhibitors THZ1, Triptolide, Flavopiridol and LCD000067 in the PTEN WT and CRISPR-*PTEN* HeLa cells, but did not detect a difference in the sensitivity of the PTEN deleted cells (Supplementary Figure S8). However, HeLa cells are genetically aberrant resulting in an activation of downstream effectors of the PI3K/AKT pathway, such as mTOR (54). Therefore, we decided to examine isogenic Pten WT and Pten^−/−^ MEFs for which we had generated microarray expression data and in which the ‘Achilles cluster’ gene set (53) was significantly enriched in Pten^−/−^ MEFs (Supplementary Figure S9). In accordance with these results, treatment of MEFs with THZ1, Triptolide, Flavopiridol or LCD000067 resulted in lower GI50s in Pten^−/−^ MEFs (Figure 5C–F; left). We observed a similar sensitivity in a pair of mouse breast tumor cell lines (MycOE Pik3ca Mut and MycOE Pten Null) and an isogenic pair of PTEN WT and PTEN^−/−^ HCT116 cell lines (55), in which the PTEN Null cells were more sensitive to transcription inhibition than the PTEN WT cells (Figure 5C–F; middle and right), implying that PTEN loss sensitizes cells to transcription inhibition.

CDK7 is an important CDK-activating kinase (CAK) during the cell cycle (56), raising the possibility that the sensitivity of PTEN Null cell lines to CDK7 inhibition is partly due to the inhibition of the cell cycle. However, there was no significant difference in the GI50s of Pten WT and Pten^−/−^ MEFs, MycOE Pik3ca Mut and MycOE Pten Null mouse breast tumor cell lines or PTEN WT and PTEN^−/−^ HCT116 cells after treatment with the cell cycle inhibitor Palbociclib (Supplementary Figure S10) (57), suggesting that loss of PTEN does not result in an increased sensitivity to cell cycle inhibition in the cell lines tested.

Next, we over-expressed wild type PTEN (PTEN WT) or phosphatase-dead PTEN C124S in MycOE Pten Null cells. While expression of PTEN WT decreased sensitivity to transcription inhibition by THZ1 or Flavopiridol (Figure 5G and H), expression of PTEN C124S did not change cells sensitivity to THZ1 treatment, and slightly increased cells sensitivity to Flavopiridol treatment (Figure 5G and H). Moreover, re-expression of PTEN WT, but not PTEN C124S decreased levels of p-AKT 473 as well as RNAPII Ser2P and Ser5P (Figure 5I). Overall, these data suggest that loss of PTEN leads to an increased sensitivity to transcription inhibition and that this effect is dependent on PTEN’s phosphatase activity.

## DISCUSSION

In this study we identified a nuclear pool of PTEN that interacts directly with AFF4 and directly or indirectly with RNAPII, CDK9, cyclin T1, XPB and CDK7, which are components of different transcription complexes. In our model, PTEN can interact with RNAPII and XPB and CDK7 during initiation and with AFF4, RNAPII, CDK9 and cyclin T1 during elongation (Supplementary Figure S11A; top). Further, we found that nuclear PTEN can bind to chromatin in overlapping regions with RNAPII, H3K4me3, and H3K27ac at promoters and with H3K27ac and H3K4me1 at putative enhancers. Notably, PTEN-bound genes have functions in key cellular processes including transcription and its regulation. Interestingly, upon loss of PTEN, levels of RNAPII Ser2P and Ser5P increase (Supplementary Figure S11A; bottom) and this is accompanied by an increased occupancy in chromatin. The changes in chromatin occupancy correlate with gene expression alterations genome-wide and more significantly at PTEN-bound genes, including for example AFF4, POLR2A, MED22, HEXIM1 and BRD2 (Supplementary Figure S11A; bottom). Moreover, loss of PTEN increased the expression of the ‘Achilles cluster’, a subset of genes, known to alter sensitivity to transcriptional inhibition (Supplementary Figure S11A; bottom) (53). Accordingly, loss of PTEN conferred sensitivity to transcription inhibition by small molecule inhibitors, such as THZ1, Triptolide, Flavopiridol, and LCD000067 in three out of four cell systems tested (Figure S11B). In summary, our findings unravel a previously unacknowledged role of nuclear PTEN in regulating RNAPII-mediated transcription and gene expression.

Despite extensive research, open questions still remain regarding the interaction partners and functions of nuclear PTEN (6). Based on data from a Y2H screen we identified AFF4, the scaffolding protein of the SEC (24) as a novel interaction partner of nuclear PTEN. As AFF4 is a binding partner of RNAPII and the P-TEFb complex during the elongation step of transcription it was not surprising to discover that RNAPII, and the P-TEFb components CDK9 and cyclin T1 (23) can interact with PTEN. It was, however, unexpected to identify XPB and CDK7, components of the TFIIH transcription factor, which is required for transcription initiation (19) as previously unappreciated interaction partners of nuclear PTEN. Based on pull down experiments using several GST-PTEN deletion constructs (full-length GST-PTEN, GST-PDC2, GST-PD, GST-C2 and GST-TAIL) we propose that the Tail-domain is dispensable for PTEN’s interactions with the transcription machinery as all but the GST-TAIL construct interacted with RNAPII, AFF4, CDK9, cyclin T1 and CDK7. This result also suggests more than one interaction interface, as both the C2-domain and the PD-domain constructs could bind to these proteins independently. However, additional experiments need to be conducted to determine the exact amino acid contacts mediating the interaction. Moreover, except for PTEN’s interaction with AFF4, which we confirmed using *in vitro* transcribed/translated AFF4, we can only speculate whether the interactions are direct or indirect. As RNAPII is a commonality between these complexes and associates with, for example, TFIIH during initiation and P-TEFb during elongation (17), we hypothesize that PTEN can bind to RNAPII throughout the different stages of the transcription cycle. However, it is also possible that chromatin or the nascent mRNA are mediators of some of these interactions and further experiments will have to be performed to address under which conditions PTEN can bind to different proteins or complexes of the transcription machinery.

Gene expression changes after loss of PTEN are well documented (12–14) and several mechanisms of how PTEN alters gene expression have been proposed (58). In agreement with previous studies, we found significant gene expression changes after loss of PTEN in HeLa cells as well as in MEFs. Furthermore, we present evidence to suggest that part of the expression changes we observed are linked to PTEN binding to the transcription machinery and chromatin. For the first time, we present genome-wide PTEN ChIP-seq data, which was validated using two different antibodies. PTEN peaks coincided with RNAPII, H3K27ac and H3K4me3 in promoter regions and H3K27ac and H3K4me1 in distal regions, indicating that PTEN can bind to DNA regulatory elements. Moreover, transcript levels of a significant number of PTEN-bound genes were altered after inactivation of PTEN. This and the overlap of a large number of PTEN and RNAPII peaks in promoter regions, suggests the binding of PTEN in concert with transcriptional complexes. Interestingly, loss of PTEN increased phosphorylation levels of RNAPII Ser2 and Ser5, and genome-wide occupancy in chromatin. The changes in chromatin occupancy correlated well with gene expression changes of differentially expressed genes due to PTEN loss and the correlation was especially strong at differentially expressed PTEN-bound genes. Notably, the differentially expressed PTEN-bound genes contained transcription machinery components as well as transcriptional regulators, such as AFF4, POLR2A, MED22, HEXIM1 and BRD2. We speculate that loss of PTEN from the promoters of these genes increases their transcript levels, which in turn affects transcription globally. An increase in transcript levels of, for example POLR2A, the CTD-containing subunit of RNAPII, might also explain the increase of RNAPII and RNAPII Ser2P and Ser5P in chromatin. Upregulation of HEXIM1 on the other hand could serve as a negative feedback loop to counteract the increase of transcriptional activators. Similarly, it has been described previously that P-TEFb can be released from its inactive complex when transcription is dramatically inhibited by UV light, actinomycin D, or P-TEFb inhibitors (59,60).

Transcription is an important universal process in non-malignant as well as malignant cells and tissues and has therefore long been considered a challenging target in cancer therapy. However, recent studies using small molecule transcriptional inhibitors have challenged this paradigm (28,53). For example, THZ1, a selective covalent inhibitor of CDK7 has been shown to effectively inhibit the growth of TNBC cell lines, in which expression of a set of genes, termed the ‘Achilles cluster’ is thought to confer sensitivity to THZ1 (53). We found the ‘Achilles cluster’ significantly enriched within PTEN-bound genes and Pten^−/−^ MEF expression data, which led us to investigate the efficacy of transcription inhibitors in PTEN-deficient cells. PTEN WT and CRISPR-*PTEN* HeLa cells were not differentially affected by treatment with transcriptional inhibitors and we speculate that this is due to additional mutations present in these cells. HeLa cells are infected with human papilloma virus (HPV) and are genetically aberrant with a deletion of the *STK11* gene, encoding the tumor suppressor LKB1 (61). HPV oncogenes and loss of LKB1 affect metabolism, cell death pathways via p53 inactivation, and growth control via Rb inactivation and have been shown to activate downstream effectors of the PI3K/AKT pathway (54).

Moreover, it has been shown that p53 activation induces transcriptional dependency to sensitize cancer cells to CDK7 inhibition (62). The relationship between PTEN and p53 is complex as the signaling pathways of p53 and PTEN are heavily intertwined (63–66). While PTEN was reported to be a downstream transcriptional target of p53 in mediating apoptosis (63) other studies suggest that PTEN may act upstream of p53 (65,66). One study found numerous p53 effectors, including p21, GDF15, PIG3, NOXA and PLK2 upregulated after PTEN deletion (67). In accordance with these results we see the GSEA hallmark gene set ‘p53_pathway’ enriched (Supplementary Figure S12A) in our Pten^−/−^ MEFs, whereas in our CRISPR-*PTEN* HeLa cells, this gene set was not enriched (Supplementary Figure S12B). As our Pten^−/−^ MEFs, but not our CRISPR-*PTEN* HeLa cells, are sensitive to transcription inhibitors, this might indicate that p53 activation contributes to the sensitivity to transcription inhibition we observe in certain cell lines after loss of PTEN.

In three cell line systems with no prior activation of the PI3K/AKT pathway we found PTEN deficient cells to be more sensitive to treatment with THZ1, Triptolide, Flavopiridol or LCD000067. Interestingly, this sensitivity could be decreased by ectopic expression of PTEN but not by phosphatase dead mutant C124S. Together these data suggest an increased sensitivity of PTEN-deficient cells to transcription inhibition, and ultimately, this could provide an effective clinical strategy to target some PTEN-deficient human cancers.

In summary, our findings indicate that in addition to its well-known role in cytoplasmic regulatory processes (58), PTEN can affect transcription in the nucleus, where through interaction with the transcription machinery in the context of chromatin, it adds an additional layer of regulatory control on RNAPII-mediated transcription.

## DATA AVAILABILITY

ChIP-seq, RNA-seq and microarray data have been deposited in the Gene Expression Omnibus (GEO) under accession number GSE120478.

ChIP-seq peaks, RNA-seq and microarray data can also be found in Supplementary Tables S2–S4.

## Supplementary Material

gkz272_Supplemental_Files

## Notes

Present address: Deepti Mathur, Computational and Systems Biology, Memorial Sloan Kettering Cancer Center, 417 E 68th St, New York, NY 10065, USA.
