# Supplementary material for: PTEN interacts with the transcription machinery on chromatin and regulates RNA polymerase II-mediated transcription
Source: Nucleic Acids Res. 2019 Apr 24;47(11):5573–86. doi: 10.1093/nar/gkz272 (PMC6582409; doi:10.1093/nar/gkz272)
Supplement: gkz272_Supplemental_Files [file gkz272_supplemental_files.zip › Supplement combined 6.4.pdf]

Description of Supplementary Figures

Figure S1: Recombinant PTEN can bind to proteins of the transcription machinery.

(A) Illustrations of full-length PTEN and AFF4 proteins. Respective fragments used in the Y2H-screen as bait- or prey-fragments are indicated.

(B) Coomassie stain of recombinant full-length GST-PTEN and GST purified from *E.coli* using glutathione sepharose beads.

(C) (left) *In vitro* translated/transcribed full-length AFF4 was incubated with recombinant GST, GST-PTEN and GST PTEN C124S sepharose beads. Pulled-down protein complexes were analyzed for AFF4 protein levels by immunoblotting. (right) Coomassie stain of recombinant GST-PTEN, GST-PTEN C124S and GST purified from *E.coli* using glutathione sepharose beads.

(D) (left) HEK293 or (right) DBTRG cell lysates were incubated with GST, GST-PTEN or control sepharose beads. Pulled-down protein complexes were analyzed for AFF4 protein levels by immunoblotting.

(E) Representative illustration of recombinant full-length GST-PTEN and GST-PTEN deletion constructs used in the experiments.

(F) Coomassie stain of recombinant full-length GST-PTEN WT and GST-PTEN deletion constructs purified from *E.coli* using glutathione sepharose beads.

(G) HEK293 cell lysates were incubated with GST, GST-PTEN, or GST-PTEN C124S sepharose beads. Pulled-down protein complexes were analyzed for AFF4, SPT5, HEXIM1, CDK9, XPB and CDK7 protein levels by immunoblotting.

Figure S2: Deletion of endogenous *PTEN* by CRISPR/Cas9.

Sequences of clones generated using CRISPR/Cas9 in HeLa cells are shown. The sequence of the guide RNA is highlighted in red.

Figure S3: Nuclear PTEN elutes with large multi-subunit complexes containing components of the transcription machinery

Size exclusion chromatography analysis of nuclear extracts from PTEN WT HeLa cells. Fractions corresponding to 2 MDa, 669 kDa and 75 kDa are indicated by black arrowheads. Protein levels of transcription machinery components were analyzed by immunoblotting.

Figure S4: PTEN binds to specific DNA motifs and loss of PTEN from promoters affects gene expression.

(A) HOMER *de novo* motif analysis was performed to identify DNA motifs bound by PTEN at (top) all peaks, (middle) promoter peaks, and (bottom) peaks in distal regions. Motifs, estimated best matches, ranks, percentages of peaks containing the motif, and p-values are given.

(B) Heatmap of (right) up- and (left) downregulated PTEN-bound genes in HeLa cells after PTEN loss. Color key is shown. Blue represents down- and red represents up-regulated genes. AFF4 and POLR2A are highlighted with red boxes.

Figure S5: PTEN loss affects RNAPII, RNAPII Ser2P and Ser5P and SPT5 occupancy at PTEN-bound genes and correlates with gene expression.

(A) Pearson correlation of log_2FC FPKM and log_2FC ChIP-Seq signals for RNAPII, RNAPII Ser2P and Ser5P and SPT5 at genes with RNAPII, RNAPII Ser2P and Ser5P or SPT5 peaks, respectively, in PTEN WT and CRISPR-*PTEN* HeLa cells. The Pearson correlation coefficients and p-values are given.

(B) (left) Schematic representation of the method used to calculate the RNAPII promoter release ratio (PRR). The promoter is defined as the region covering 100 bp upstream to 300 bp downstream of the TSS and the gene body is defined as the region from 300 bp to 2 kb downstream of the TSS. (right) Comparison of the RNAPII PPR calculated in PTEN WT and CRISPR-*PTEN* HeLa cells. Tukey boxplots with outliers omitted. Student's t-test, ns, non-significant.

(C) Pearson correlation of log_2FC FPKM and log_2FC ChIP-Seq signals for RNAPII, RNAPII Ser2P and Ser5P and SPT5 at PTEN-bound genes in PTEN WT and CRISPR-*PTEN* HeLa cells. The Pearson correlation coefficients and p-values are given.

Figure S6: PTEN loss affects RNAPII occupancy in chromatin in MEFs and MycOE mouse breast tumor cells.

(A) RNAPII ChIP-seq in Pten WT (blue) and $Pten^{-/-}$ (red) MEFs. Plots represent average read counts per 50 bp bins. (Top left) ChIP-Seq meta-profiles. Gene bodies are scaled to 5 kb and 3 kb up- and 5 kb downstream are shown. (Top right) ChIP-Seq meta-profiles. Peaks are centered around the TSS and ±1 kb are shown. (Bottom left) Data for heatmaps is rank-ordered according to occupancy in Pten WT MEFs. (Bottom right)

Log_{10}intensity of ChIP-seq signal in gene bodies. Tukey boxplots with outliers omitted. Student's t-test, **$p \leq 0.01$.

(B) RNAPII ChIP-seq in MycOE Pik3ca Mut (blue) and MycOE Pten Null (red) cells. Plots represent average read counts per 50 bp bins. (Top left) ChIP-Seq meta-profiles. Gene bodies were scaled to 5 kb and 3 kb up- and 5 kb downstream are shown. (Top right) ChIP-Seq meta-profiles. Peaks are centered around the TSS and ±1 kb are shown. (Bottom left) Data for heatmaps is rank-ordered according to occupancy in MycOE Pik3ca Mut cells. (Bottom right) Log_{10}intensity of ChIP-seq signal in gene bodies. Tukey boxplots with outliers omitted. Student's t-test, ****$p \leq 0.0001$.

(C) Pearson correlation of log_2FC FPKM and log_2FC ChIP-Seq signal for RNAPII in Pten WT and $Pten^{-/-}$ MEFs. The Pearson correlation coefficient and p-value are given.

Figure S7: PTEN WT can rescue the transcription activation seen in a subset of genes after loss of PTEN.

RT–qPCR analysis in PTEN WT and CRISPR-*PTEN* HeLa overexpressing empty control plasmid (EV) or wild type PTEN (PTEN WT). Error bars represent standard deviations (n = 3). Data were analyzed using the delta-delta-Ct method and were normalized to the PTEN WT OE-EV sample. Student's t-test, *$p \leq 0.05$.

Figure S8: PTEN loss does not sensitize HeLa cells to transcription inhibition.

GI50 values of PTEN WT and CRISPR-*PTEN* HeLa cells treated with dose titrations of THZ1, Triptolide, Flavopiridol or LCD000067. Data are presented as mean ±SD (n = 3). Student's t-test, *$p < 0.05$; ns, non-significant.

Figure S9: Pten loss upregulates THZ1-sensitive genes in MEFs.

GSEA of the “Achilles cluster” gene set (1) in Pten WT and Pten$^{-/-}$ MEFs. Red color represents positive correlation, blue color represents negative correlation. GSEA-supplied normalized enrichment score (NES), nominal p-value, and the false discovery rate q-value (FDR q-value) are shown.

Figure S10: PTEN status does not confer sensitivity to Palbociclib treatment.

GI50 values of Pten WT and Pten$^{-/-}$ MEFs, MycOE Pik3ca Mut and MycOE Pten Null cells, and PTEN WT and PTEN$^{-/-}$ HCT116 cells treated with dose titrations of Palbociclib. Data are presented as mean ±SD (n = 3). Student’s t-test, ns, non-significant.

Figure S11: PTEN binds to transcription complexes and mediates sensitivity to transcriptional inhibition by small molecule inhibitors.

(A) In the model PTEN can interact with transcription complexes containing XPB, CDK7 and RNAPII during initiation and complexes containing AFF4, CDK9, cyclin T1, and RNAPII during elongation. Upon loss of PTEN levels of RNAPII Ser2P and Ser5P increase and this correlates with gene expression changes, for example the upregulation of genes involved in transcription and within the “Achilles cluster” gene set.

(B) PTEN-deficient cells show an increased sensitivity to transcription inhibition.

Figure S12: PTEN loss leads to the enrichment of the GSEA hallmark gene set “p53_pathway” in cells without prior PI3K/AKT-pathway activation.

GSEA of the “Hallmark_p53_pathway” gene set (1) in (A) Pten WT and Pten$^{-/-}$ MEFs and (B) PTEN WT and CRISPR-*PTEN* HeLa cells. Red color represents positive correlation, blue color represents negative correlation. GSEA-supplied normalized enrichment score (NES), nominal p-value, and the false discovery rate q-value (FDR q-value) are shown.

Description of Supplementary Tables

Table S1: Yeast-2-hybrid screen data

Related to Figures 2 and S1.

The Y2H performed by Hybrigenics Services identified 46 peptides as potential interactors of PTEN. A portion of PTEN was used as the bait fragment against a library of prey-fragments of the human fetal brain.

Table S2: HeLa RNA-seq data

Related to Figures 3, 4, 5, S4 and S5.

Tab 1: Number of reads for each sample after processing. Tab 2: RNA-seq data of three PTEN WT and three CRISPR-*PTEN* HeLa clones. Tab 3: 1540 genes were differentially expressed after PTEN loss from HeLa cells. Tab 4: 300 genes were differentially expressed and PTEN-bound (promoters and distal regions). Values are normalized and presented as FPKM.

Table S3: MEF microarray data

Related to Figures S6 and S9.

Microarray data of four isogenic pairs of Pten WT and $Pten^{-/-}$ MEFs. Values were normalized using the Affymetrix package.

Table S4: ChIP-seq genomic data

Related to Figures 3, 4, S4 and S5.

Tab 1: PTEN ChIP-seq peaks (significance cut-off -log10(q-value)>5) called with MACS2 that were present in two ChIP experiments using two different PTEN antibodies; each peak is presented with UCSC GRCh37/hg19 coordinates and its unique ID. Tabs 2 and 3: PTEN ChIP-seq peaks (significance cut-off -log10(q-value)>5) called with MACS2 that fell within or outside 1 kb of a TSS; each peak is presented with UCSC GRCh37/hg19 coordinates, its unique ID and associated gene. Tabs 4–7: RNAPII, RNAPII Ser2P, RNAPII Ser5P and SPT5 ChIP-seq significant peaks in PTEN WT and CRISPR-*PTEN* HeLa cell lines called with MACS2; each peak is presented with UCSC GRCh37/hg19 coordinates, its unique ID and associated gene. Tabs 8 and 9: RNAPII ChIP-seq significant peaks in Pten WT and Pten$^{-/-}$ MEFs or MycOE Pik3ca and MycOE Pten Null cell lines called with MACS2; each peak is presented with UCSC NCBI37/mm9 coordinates, its unique ID and associated gene.

Table S5: Sequencing information for ChIP-seq

Related to Supplemental Methods.

The final numbers of reads after processing are shown for each ChIP-seq sample.

Supplementary Materials and Methods

Antibodies

Protein Target	Purpose	Company	Catalogue #	Type	Concentration
actin	WB	Sigma-Aldrich	A4700	mouse monoclonal	1:5000
AFF4	WB	abcam	ab57077	mouse monoclonal	1:1000
p-AKT (Ser473)	WB	Cell Signaling Technology	3787	rabbit monoclonal	1:1000
BRD4	WB	Bethyl Laboratories	A301-985A	rabbit polyclonal	1:1000
p-BRD4 (484/488)	WB	Gift from C.M. Chiang	na	rabbit polyclonal	1:1000
CDK7 (MO1)	WB	Cell Signaling Technology	2916	mouse monoclonal	1:1000
CDK9	WB	Santa Cruz	sc-484	rabbit polyclonal	1:1000
CDK9	WB, PLA	Santa Cruz	sc-484-G	goat polyclonal	1:1000, 1:100
cyclin H	WB	abcam	ab92376	Rabbit polyclonal	1:1000
cyclin T1	WB, PLA	Santa Cruz	sc-10750	rabbit polyclonal	1:1000, 1:100
H3	WB	abcam	ab1791	rabbit polyclonal	1:10000
HEXIM1	WB	abcam	ab25388	rabbit polyclonal	1:1000
PTEN (138G6)	PLA, ChIP-seq	Cell Signaling Technology	9559	rabbit monoclonal	1:100, 5 ug/ChIP
PTEN (6H2.1)	WB, ChIP-seq	EMD Millipore	04-035	mouse monoclonal	1:1000, 5 ug/ChIP
p-PTEN (380/382/383)	WB	Cell Signaling Technology	9549	rabbit monoclonal	1:1000
RNAPII (8WG16)	WB	abcam	ab817	mouse monoclonal	1:1000
RNAPII (N-20)	ChIP-seq	Santa Cruz	Sc-899	rabbit polyclonal	5 ug/ChIP
RNAPII Ser2P	WB, ChIP-seq	abcam	ab5095	rabbit polyclonal	1:5000, 5 ug/ChIP
RNAPII Ser5P	WB, ChIP-seq	abcam	ab5408	mouse monoclonal	1:5000, 5 ug/ChIP
SPT5	WB, ChIP-seq	Bethyl Laboratories	A300-868A	rabbit polyclonal	1:1000, 5 ug/ChIP
p-SPT5 (806)	WB	Gift from R.P. Fisher	na	Rabbit polyclonal	1:1000
XPB	WB	Bethyl Laboratories	A301-337A-T	rabbit polyclonal	1:1000
V5	WB	Invitrogen	R960-25	mouse monoclonal	1:2000
vinculin	WB	Sigma-Aldrich	V9131	mouse monoclonal	1:5000

Primers

Primers used for sequence analysis of CRISPR-*PTEN* clones:

Forward: TGATGGGAAAATGATGTCTGA

Reverse: ATGGGCTCAAATATGGGCTA

Immunofluorescence

WT and $Pten^{-/-}$ MEFs were plated in complete MEF medium on gelatin coated glass cover slips, fixed in 2% paraformaldehyde, permeablized in perm/block solution (10% goat serum, 0.1% Triton X-100 in PBS) and incubated with the primary antibody (PTEN 138G6, 1:200) overnight at 4°C. Slides were incubated with TRITC-conjugated goat anti-rabbit secondary antibody (Jackson ImmunoResearch, 1:500), mounted using ProLong® Gold Antifade Mountant with DAPI (Invitrogen) and imaged using a Nikon Eclipse N*i* microscope at 20x magnification.

Cell fractionation

Cell fractionation was performed as described before (2). In brief, cells were lysed in buffer II (0.32 M Sucrose, 60 mM KCl, 5 mM $MgCl_2$, 0.1 mM EDTA, 15 mM Tris pH 7.5, 0.5 mM DTT, 1x protease inhibitor and 0.04% IGEPAL-600) and layered on top of a sucrose cushion (1.2 M Sucrose, 60 mM KCl, 5 mM $MgCl_2$, 0.1 mM EDTA 15 mM Tris pH 7.5, 0.5 mM DTT, 1x protease inhibitor). Nuclei were pelleted at 10,000 x g for 20 min at 4°C and resuspended in Buffer A (0.32 M Sucrose, 50 mM Tris pH 7.5, 4 mM $MgCl_2$, 1 mM $CaCl_2$, 0.5 mM DTT, 1x protease inhibitor). Micrococcal nuclease digestion was performed in Buffer A supplemented with 2 mM $CaCl_2$ using 0.06 units of

micrococcal nuclease per 1 µg of chromatin. The chromatin was pelleted at 1000 x g for 7 min at 4ºC and resuspended in 10 mM EDTA. Samples were incubated with 0.5 M NaCl for 45 min rotating and centrifuged at full speed for 5 min at 4°C.

GST fusion protein purification

Plasmids were transformed into BL21(DE3) pLysE chemically competent cells (Invitrogen). Cells were grown until OD600 0.3-0.5 and protein expression was induced with 0.1 mM Isopropyl β-D-1-thiogalactopyranoside for 16 h at 21°C. The bacteria were pelleted and the pellet was resuspended in phosphatase buffer (25 mM Tris pH 7.4, 100 mM NaCl). The lysate was sonicated for 6 min (30s on, 30 off intervals), precleared and incubated overnight with glutathione sepharose beads rotating at 4°C (GE Healthcare Life Sciences). Beads were washed with phosphatase buffer, resuspended with the bead bed volume of phosphatase buffer, snap frozen and stored at -80ºC.

GST bead pull-downs

HEK293 or DBTRG cells were rinsed with PBS and lysed in BC200 (25 mM Tris pH 7.5, 200 mM NaCl, 1 mM EDTA, 0.2% Triton X-100, 0.2% Glycerol). Lysates were sonicated, centrifuged, and then pre-cleared with glutathione sepharose beads (GE Healthcare Life Sciences) for 1 h rotating at 4°C. Supernatants were incubated with GST-PTEN or indicated GST-PTEN domains loaded onto glutathione sepharose beads rotating at 4°C overnight. Beads were washed eight times with BC200 and proteins were eluted with the elution buffer (25 mM TRIS pH 8.0, 150 mM NaCl, 50 mM glutathione).

In vitro transcription/translation assay

In vitro transcription/translation assays were performed according to the manufacturer's protocol (TnT® Quick Coupled Transcription/Translation System, Promega) using PCR-generated full-length AFF4 as the template.

Size exclusion chromatography

Nuclear extracts from $3x10^8$ HeLa cells were prepared following a slightly modified Dignam-Roeder protocol (3). ~$3x10^8$ cells were scraped into PBS and pelleted. Cells were resuspended in five times packed cell volume (pcv) of Dignam hypotonic buffer (10 mM HEPES-KOH pH 7.9, 10 mM KCl, 1.5 mM $MgCl_2$, 0.5 mM DTT, 1x protease inhibitor cocktail), pelleted and incubated on ice for 10 min in three times pcv of Dignam hypotonic buffer. Cells were homogenized, nuclei were pelleted, and resuspended in 1 time packed nuclei volume (pnv) of Dignam high Salt buffer (20 mM HEPES-KOH pH 7.9, 350 mM NaCl, 1.5 mM $MgCl_2$, 0.2 mM EDTA, 25% glycerol, 0.5 mM DTT, 1x protease inhibitor cocktail). Nuclear extracts were incubated for at least 1 h rotating at 4ºC and cleared by centrifugation before application to size exclusion chromatography. 1 mL of nuclear extract was loaded onto a size exclusion chromatography column (Superose 6 10/300 Increase GL, GE Healthcare) connected to an ÄKTA Pure system. The column was equilibrated with size-exclusion buffer (40 mM HEPES pH 7.5, 350 mM NaCl, 10% glycerol, and 0.1% Tween 20). Proteins were eluted with 1.5 column volumes of size exclusion buffer and analyzed by immunoblotting.

RNA Microarrays

250 ng of RNA per sample from 4 isogenic pairs of Pten WT and $Pten^{-/-}$ MEFs were collected 4 passages after infection with Adeno-Cre containing virus and were processed according to the Ambion® Whole Transcript (WT) Expression Array (Life Technologies) manufacturer's protocol. Samples were analyzed by Applied Biosystems using GeneChips Mouse Gene 2.0 ST Arrays (Affymetrix). Results were imported into R for quality control (including consistency of probe hybridization and signal intensity) and normalization was performed using the Affymetrix package.

Chromatin Immunoprecipitation (ChIP)

One-step and two-step crosslinking ChIP experiments were performed as described before (4,5).

One-step crosslinking protocol

After cross-linking using 1% formaldehyde for 10 min at RT, cells were scraped off the plates and pelleted by centrifugation. To prepare samples for sonication, cells were swelled on ice for 10 min (25 mM Hepes pH 7.8, 10 mM KCl, 1.5 mM $MgCl_2$ and added fresh 0.1% NP-40 1 mM DTT, 1x protease inhibitor), nuclei were isolated and resuspended in sonication buffer (50 mM Hepes pH 7.9, 140 mM NaCl, 1 mM EDTA, 1% Triton X-100 and added fresh 0.1% sodiumdeoxycholate, 1% SDS, 1x protease inhibitor) and subjected to sonication in 15 mL falcon tubes using the UCD-400 Bioruptor (Diagenode) for 20-40 cycles (30s on/30s off).

After sonication, samples were pre-cleared by centrifugation and the SDS concentration was adjusted to 0.1% by addition of sonication buffer without SDS. Magna ChIP Protein A+G Magnetic Beads (Millipore) were added for 2h and the pre-cleared samples were incubated with 5 µg of RNAPII antibody (N-20, Santa Cruz) overnight at 4ºC. Magna ChIP Protein A+G Magnetic Beads were added and the beads were washed once with each of the following buffers: Low salt wash buffer (0.1% SDS, 1% Triton X-100, 2 mM EDTA, 20 mM Tris-HCl, pH 8.1, 150 mM NaCl); High salt wash buffer (0.1% SDS, 1% Triton X-100, 2 mM EDTA, 20 mM Tris-HCl, pH 8.1, 500 mM NaCl); LiCl wash buffer (0.25 M LiCl, 1% deoxycholate, 1% NP40, 1 mM EDTA, 10 mM Tris-HCl, pH 8.1); TE buffer (10 mM Tris-HCl, pH 8.1, 1 mM EDTA).

Two-step crosslinking protocol

Crosslinking was performed using 2 mM Disuccinimidyl glutarate (Sigma-Aldrich) for 45 min at RT, followed by 1% formaldehyde for 10 min at RT. Cells were then scraped and pelleted by centrifugation. For sonication cells were resuspended in Cell Lysis Buffer (10 mM Tris pH 8, 10 mM NaCl, 0.2% NP-40, 1 mM PMSF, 1:1000 protease inhibitor cocktail) and incubated on ice for 15 min. Nuclei were isolated and resuspended in Nuclear Lysis Buffer (50 mM Tris pH 8, 10 mM EDTA, 1% SDS, 1 mM PMSF, 1:1000 protease inhibitor cocktail), incubated on ice for 10 min and subjected to sonication in Eppendorf tubes using the UCD-400 Bioruptor (Diagenode) for 12-15 cycles (30s on/30s off). Samples were pre-cleared by centrifugation and diluted 1:4 with IP Dilution buffer (20mM Tris pH 8, 2 mM EDTA, 150 mM NaCl, 1% Triton-X, 0.01% SDS, 1 mM PMSF, 1:1000 protease inhibitor cocktail). Chromatin was further pre-cleared with

Magna ChIP A+G (Millipore) magnetic beads pre-conjugated with rabbit or mouse IgG respectively. Beads were removed and the supernatant was incubated with pre-conjugated antibodies overnight at 4°C. Beads were washed 2x with High Salt Buffer (20mM Tris pH 8, 2 mM EDTA, 500 mM NaCl, 1% Triton-X, 0.01% SDS, 1 mM PMSF, 1:1000 protease inhibitor cocktail), 1x with IP Wash I (20 mM Tris pH 8, 2 mM EDTA, 50 mM NaCl, 1% Triton-X, 0.1% SDS, 1 mM PMSF, 1:1000 protease inhibitor cocktail), 1x with IP Wash II (10 mM Tris pH 8, 1 mM EDTA, 0.25 LiCl, 1% NP-40, 1% deoxycholic acid, 1 mM PMSF, 1:1000 protease inhibitor cocktail), and 2x with TE (5 mM Tris pH 7.4, 1 mM EDTA).

To elute the chromatin, beads were incubated twice with elution buffer (1% SDS, 0.1 M sodium bicarbonate) by shaking on a thermomixer for 30 min at 65ºC. To reverse-crosslink the chromatin, 300 mM NaCl was added, and the sample was incubated at 65°C overnight. The sample was then treated with RNase for 30 min at 37ºC, and proteinase K for 2 h at 45ºC. The DNA was extracted using the MinElute PCR Purification Kit (Qiagen) and analyzed using a High Sensitivity ChIP on the Agilent 2100 Bioanalyzer.

ChIP-seq library preparation and sequencing

ChIP-seq libraries were generated as described before (6). Briefly, 2-10 ng of DNA were subject to end-repair, A-tailing, and ligation with Illumina Truseq adapters using NEB enzymes (NEB), followed by size-selection of 300-400 bp and amplification for 10-15 cycles using the KAPA HiFi Library Amplification Kit (Kapa Biosystems). Libraries were purified, quantified, multiplexed and submitted to 75 bp single-end sequencing using the

NextSeq® 500/550 High output Kit v2 (75 cycles) on a NextSeq® 500 sequencing system (Illumina).

ChIP-seq analysis

Alignment

FastQC was run on the ChIP-seq raw data for quality control. The Bowtie short read aligner (version 1.1.1) (7) was used to align reads to the genome (GRCh37/hg19 for HeLa samples, NCBI37/mm9 for MEF and MycOE mouse breast samples). The following settings were used (if not specified, the default settings were applied): a seed length of 50 bp (-l 50) was used, and only the single (-m 1) best alignment (--best) per read with up to two mismatches (-n 2) was reported in the SAM file.

Peak calling

MACS2: Significant peak calling was performed using MACS2 (version 2.1.0) (8). The following options were used (if not specified, the default settings were applied): --nomodel, --extsize 150 and --keep-dup 2. Pileups were generated using MACS2 (Fold enrichment over Input control, --SPMR option).

Peak annotation

The BEDtools suite (version v2.21.0) (9) was used to assign called peaks to the most proximal transcript.

Data visualization

Wiggle files for gene tracks were uploaded to the UCSC genome browser *(http://genome.ucsc.edu)* (10).

Plots for average fold enrichment patterns and heat maps were generated using Galaxy/deepTools 2 (11).

Promoter release ratio

The promoter release ration (PRR) was calculated by taking the average coverage in the promoter region (-100 bp to +300 bp of the TSS) divided by the average coverage of the gene body (+300 bp to +2 kb of the TSS).

Supplemental References

1. Wang, Y., Zhang, T., Kwiatkowski, N., Abraham, B.J., Lee, T.I., Xie, S., Yuzugullu, H., Von, T., Li, H., Lin, Z. *et al.* (2015) CDK7-dependent transcriptional addiction in triple-negative breast cancer. *Cell*, **163**, 174-186.
2. Umlauf, D., Goto, Y. and Feil, R. (2004) Site-specific analysis of histone methylation and acetylation. *Methods Mol Biol*, **287**, 99-120.
3. Dignam, J.D., Lebovitz, R.M. and Roeder, R.G. (1983) Accurate transcription initiation by RNA polymerase II in a soluble extract from isolated mammalian nuclei. *Nucleic Acids Res*, **11**, 1475-1489.
4. Niu, H., Cattoretti, G. and Dalla-Favera, R. (2003) BCL6 controls the expression of the B7-1/CD80 costimulatory receptor in germinal center B cells. *J Exp Med*, **198**, 211-221.
5. Tian, B., Yang, J. and Brasier, A.R. (2012) Two-step cross-linking for analysis of protein-chromatin interactions. *Methods Mol Biol*, **809**, 105-120.
6. Hasson, D., Panchenko, T., Salimian, K.J., Salman, M.U., Sekulic, N., Alonso, A., Warburton, P.E. and Black, B.E. (2013) The octamer is the major form of CENP-A nucleosomes at human centromeres. *Nat Struct Mol Biol*, **20**, 687-695.
7. Langmead, B., Trapnell, C., Pop, M. and Salzberg, S.L. (2009) Ultrafast and memory-efficient alignment of short DNA sequences to the human genome. *Genome Biol*, **10**, R25.
8. Zhang, Y., Liu, T., Meyer, C.A., Eeckhoute, J., Johnson, D.S., Bernstein, B.E., Nusbaum, C., Myers, R.M., Brown, M., Li, W. *et al.* (2008) Model-based analysis of ChIP-Seq (MACS). *Genome Biol*, **9**, R137.
9. Quinlan, A.R. and Hall, I.M. (2010) BEDTools: a flexible suite of utilities for comparing genomic features. *Bioinformatics*, **26**, 841-842.
10. Kent, W.J., Sugnet, C.W., Furey, T.S., Roskin, K.M., Pringle, T.H., Zahler, A.M. and Haussler, D. (2002) The human genome browser at UCSC. *Genome Res*, **12**, 996-1006.
11. Ramirez, F., Dundar, F., Diehl, S., Gruning, B.A. and Manke, T. (2014) deepTools: a flexible platform for exploring deep-sequencing data. *Nucleic Acids Res*, **42**, W187-191.

Figure S1

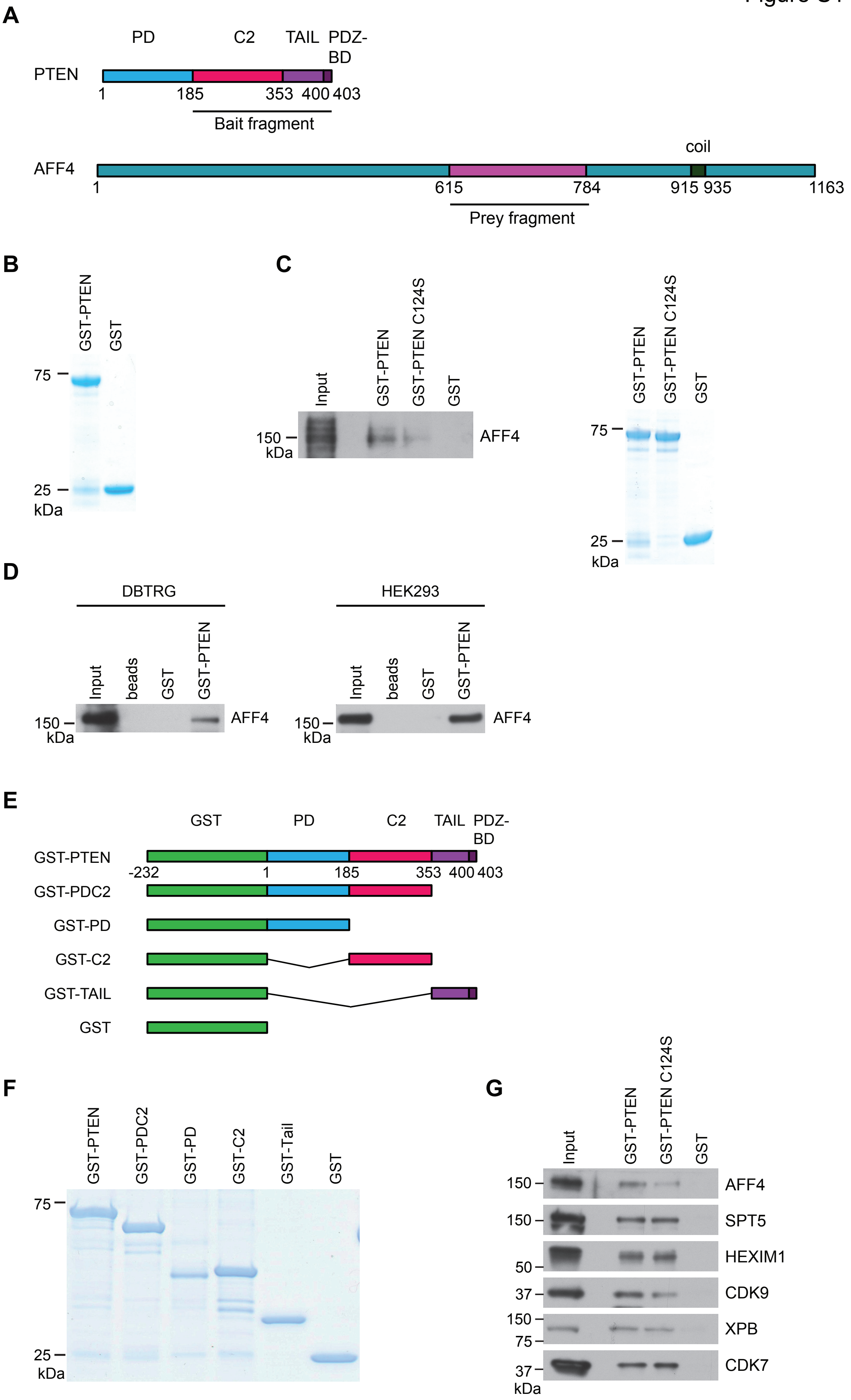
A
PD
C2
TAIL
PDZ-BD
PTEN
1
185
353
400
403
Bait fragment
coil
AFF4
1
615
784
915
935
1163
Prey fragment
B
GST-PTEN
GST
75
25
kDa
C
Input
GST-PTEN
GST-PTEN C124S
GST
150
kDa
AFF4
GST-PTEN
GST-PTEN C124S
GST
75
25
kDa
D
DBTRG
Input
beads
GST
GST-PTEN
150
kDa
AFF4
HEK293
Input
beads
GST
GST-PTEN
150
kDa
AFF4
E
GST
PD
C2
TAIL
PDZ-BD
GST-PTEN
-232
1
185
353
400
403
GST-PDC2
GST-PD
GST-C2
GST-TAIL
GST
F
GST-PTEN
GST-PDC2
GST-PD
GST-C2
GST-Tail
GST
75
25
kDa
G
Input
GST-PTEN
GST-PTEN C124S
GST
150
AFF4
150
SPT5
50
HEXIM1
37
CDK9
150
75
XPB
37
CDK7
kDa

Figure S2

WT 5’-GGATTCAAAGCATAAAAACCATTACAAGATATACAATCTGTAAGTATGTTTTCTTATTTG-3’

Clone 1
Allele 1 5’-GGATTCAAAGCATAAAAACCA---------ATACAATCTGTAAGTATGTTTTCTTATTTG-3’
Allele 2 5’-GGATTCAAAGCATAAAAACC-----AAGATATACAATCTGTAAGTATGTTTTCTTATTTG-3’

Clone 2
Allele 1 5’-GGATTCAAAGCATAAA------------------- ATCTGTAAGTATGTTTTCTTATTTG-3’
Allele 2 5’-GGATTCAAAGCATAAAAACCATTA---------CAATCTGTAAGTATGTTTTCTTATTTG-3’

Figure S3

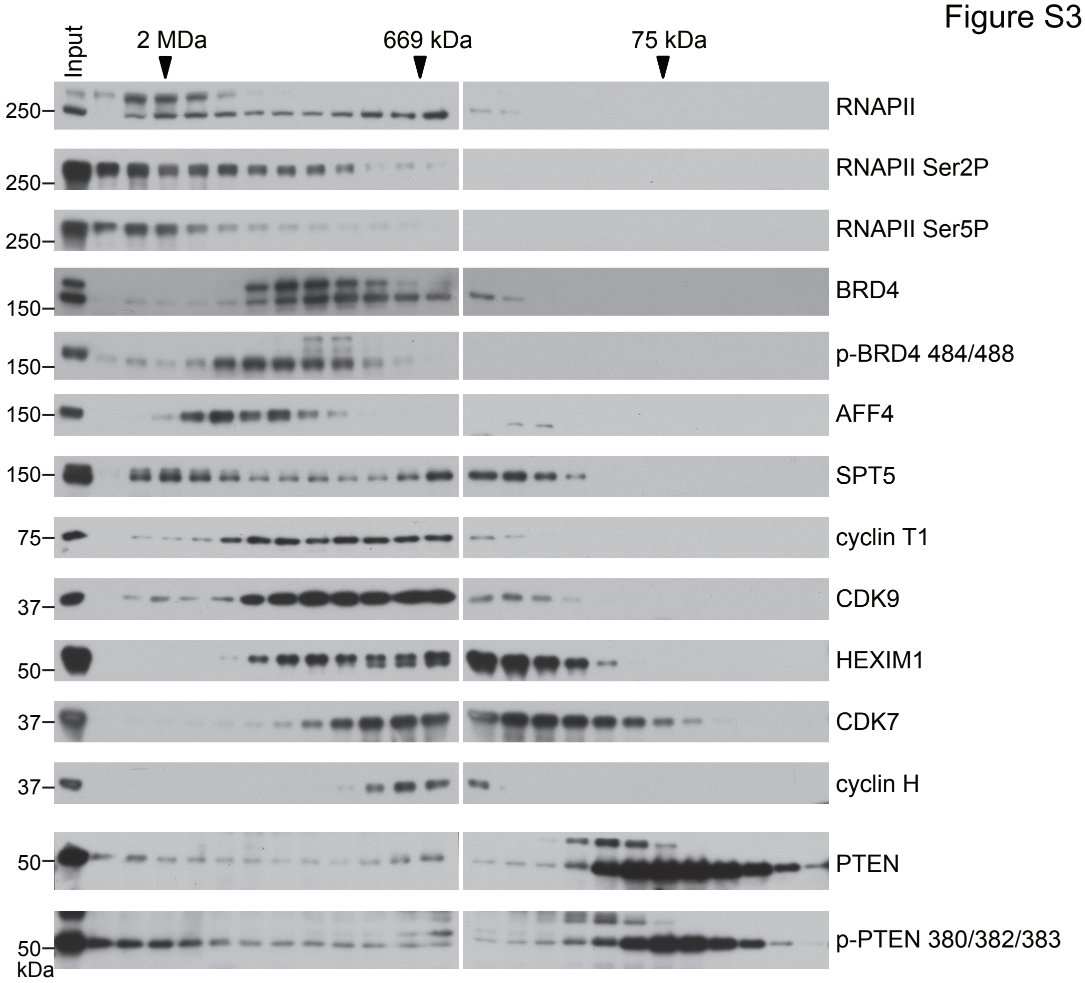

Input
2 MDa
669 kDa
75 kDa
250–
RNAPII
250–
RNAPII Ser2P
250–
RNAPII Ser5P
150–
BRD4
150–
p-BRD4 484/488
150–
AFF4
150–
SPT5
75–
cyclin T1
37–
CDK9
50–
HEXIM1
37–
CDK7
37–
cyclin H
50–
PTEN
50–
kDa
p-PTEN 380/382/383

Figure S4

A

All PTEN peaks

Motif	Best Match	Rank	% of sites	p-value
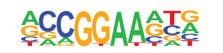	Elk1	1	28.8	1e-108
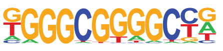	Sp1	2	30.1	1e-891
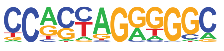	BORIS-CTCFL	3	5.8	1e-79
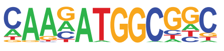	YY1	4	7.4	1e-75

PTEN peaks in promoter regions

Motif	Best Match	Rank	% of sites	p-value
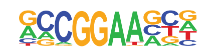	Elk4	1	31.7	1e-114
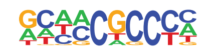	RNAPII GC-box	2	38.6	1e-81
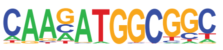	YY1	3	9.0	1e-71

PTEN peaks in distal regions

Motif	Best Match	Rank	% of sites	p-value
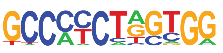	BORIS/CTCFL	1	15.8	1e-96
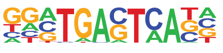	Fra1	2	15.4	1e-63

B

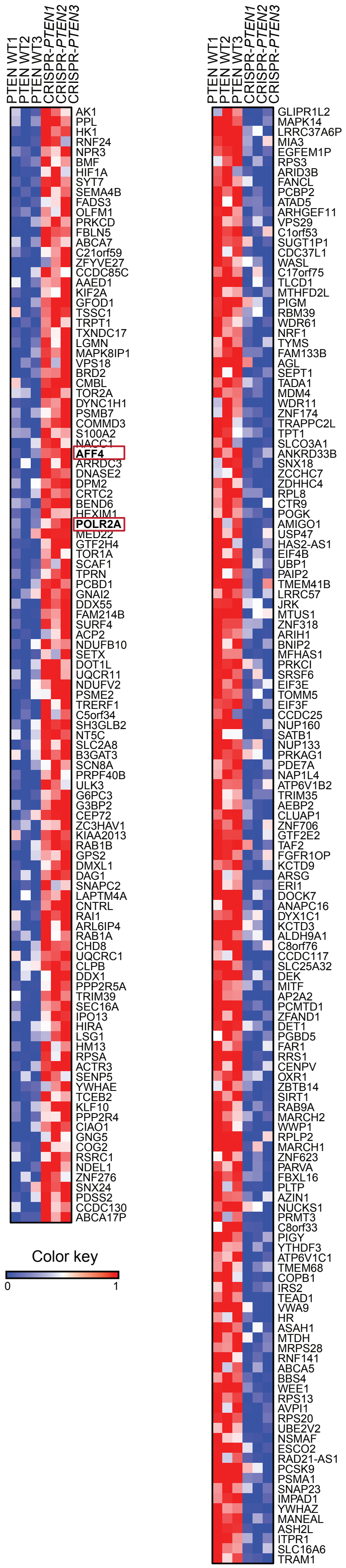

Figure S5

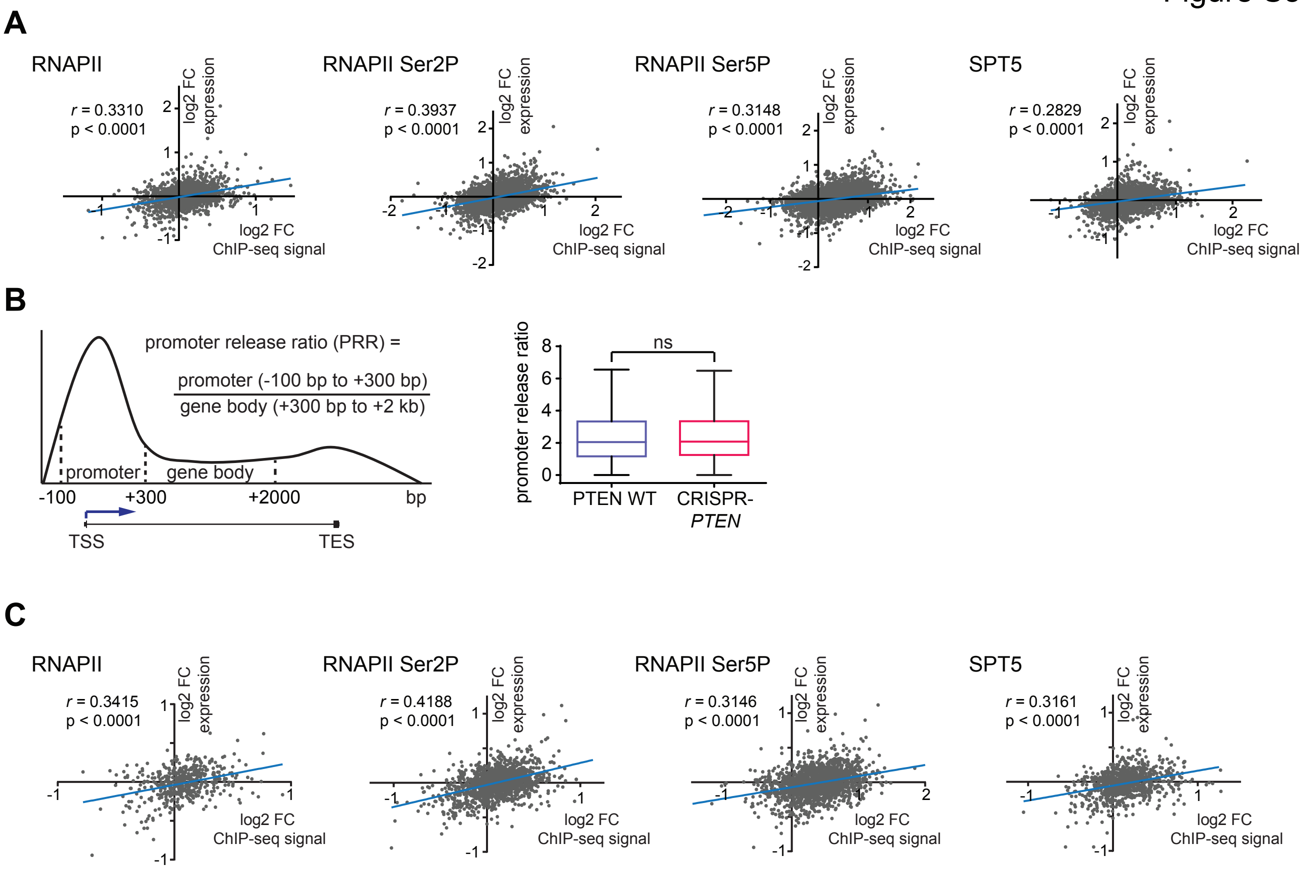
A
RNAPII
r = 0.3310
p < 0.0001
RNAPII Ser2P
r = 0.3937
p < 0.0001
RNAPII Ser5P
r = 0.3148
p < 0.0001
SPT5
r = 0.2829
p < 0.0001
log2 FC expression
log2 FC ChIP-seq signal
B
promoter release ratio (PRR) =
promoter (-100 bp to +300 bp)
gene body (+300 bp to +2 kb)
promoter
gene body
-100
+300
+2000
bp
TSS
TES
promoter release ratio
ns
PTEN WT
CRISPR-
PTEN
C
RNAPII
r = 0.3415
p < 0.0001
RNAPII Ser2P
r = 0.4188
p < 0.0001
RNAPII Ser5P
r = 0.3146
p < 0.0001
SPT5
r = 0.3161
p < 0.0001
log2 FC expression
log2 FC ChIP-seq signal

Figure S6

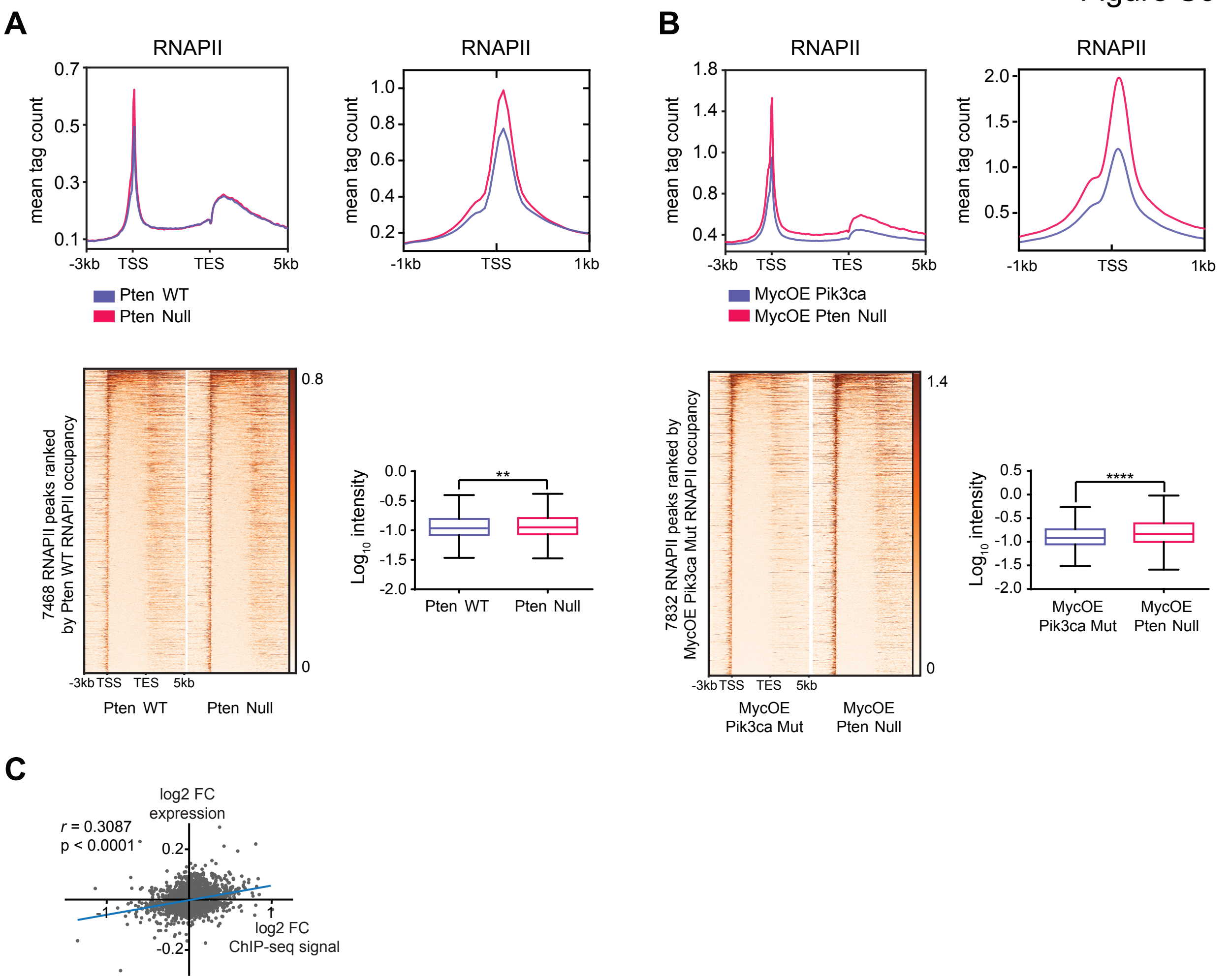
A
RNAPII
mean tag count
-3kb TSS TES 5kb
-1kb TSS 1kb
Pten WT
Pten Null
7468 RNAPII peaks ranked by Pten WT RNAPII occupancy
Pten WT Pten Null
Log10 intensity
**
B
RNAPII
MycOE Pik3ca
MycOE Pten Null
7832 RNAPII peaks ranked by MycOE Pik3ca Mut RNAPII occupancy
MycOE Pik3ca Mut
MycOE Pten Null
****
C
log2 FC expression
r = 0.3087
p < 0.0001
log2 FC ChIP-seq signal

Figure S7

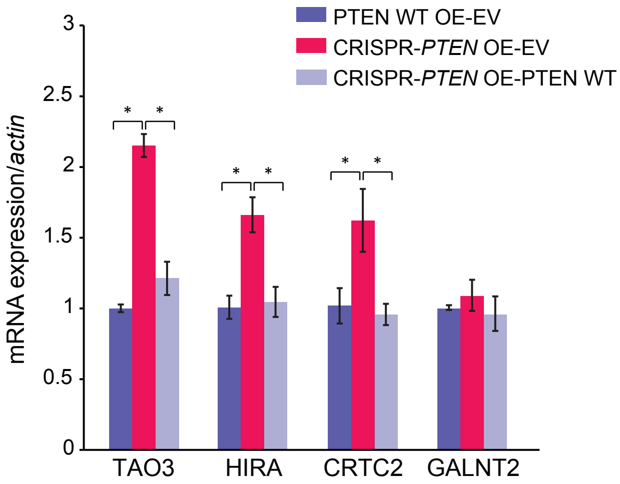
PTEN WT OE-EV
CRISPR-*PTEN* OE-EV
CRISPR-*PTEN* OE-PTEN WT
mRNA expression/*actin*
0
0.5
1
1.5
2
2.5
3
TAO3
HIRA
CRTC2
GALNT2

Figure S8

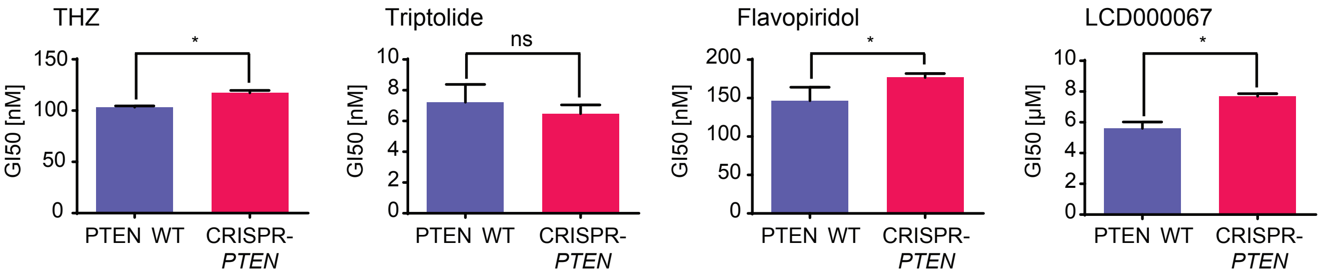
THZ
*
150
100
50
0
GI50 [nM]
PTEN WT
CRISPR-PTEN
Triptolide
ns
10
8
6
4
2
0
GI50 [nM]
PTEN WT
CRISPR-PTEN
Flavopiridol
*
200
150
100
50
0
GI50 [nM]
PTEN WT
CRISPR-PTEN
LCD000067
*
10
8
6
4
2
0
GI50 [µM]
PTEN WT
CRISPR-PTEN

Figure S9

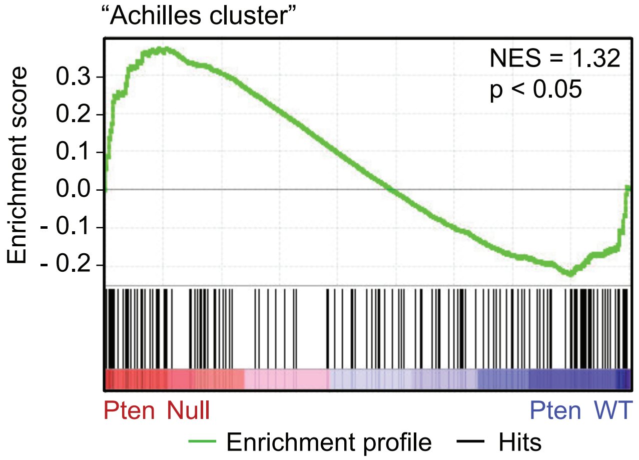

Normalized enrichment score = 1.32
Nominal p-value = 0.042
FDR q-value = 0.026

Figure S10

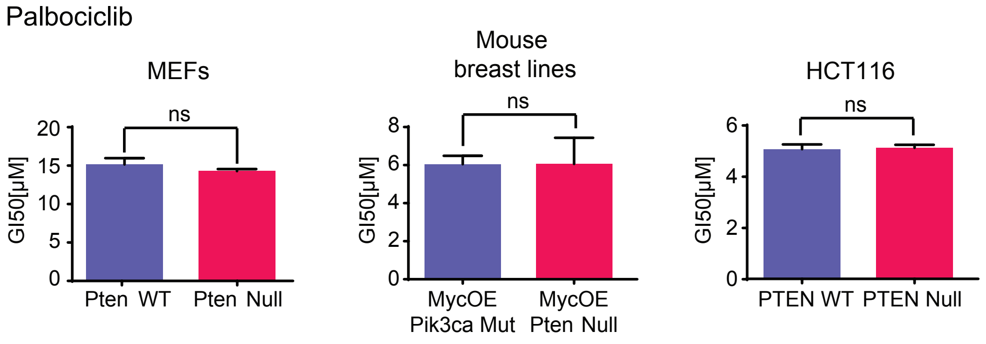
Palbociclib
MEFs
ns
20
15
10
5
0
GI50[µM]
Pten WT
Pten Null
Mouse
breast lines
ns
8
6
4
2
0
GI50[µM]
MycOE
Pik3ca Mut
MycOE
Pten Null
HCT116
ns
6
4
2
0
GI50[µM]
PTEN WT
PTEN Null

Figure S11

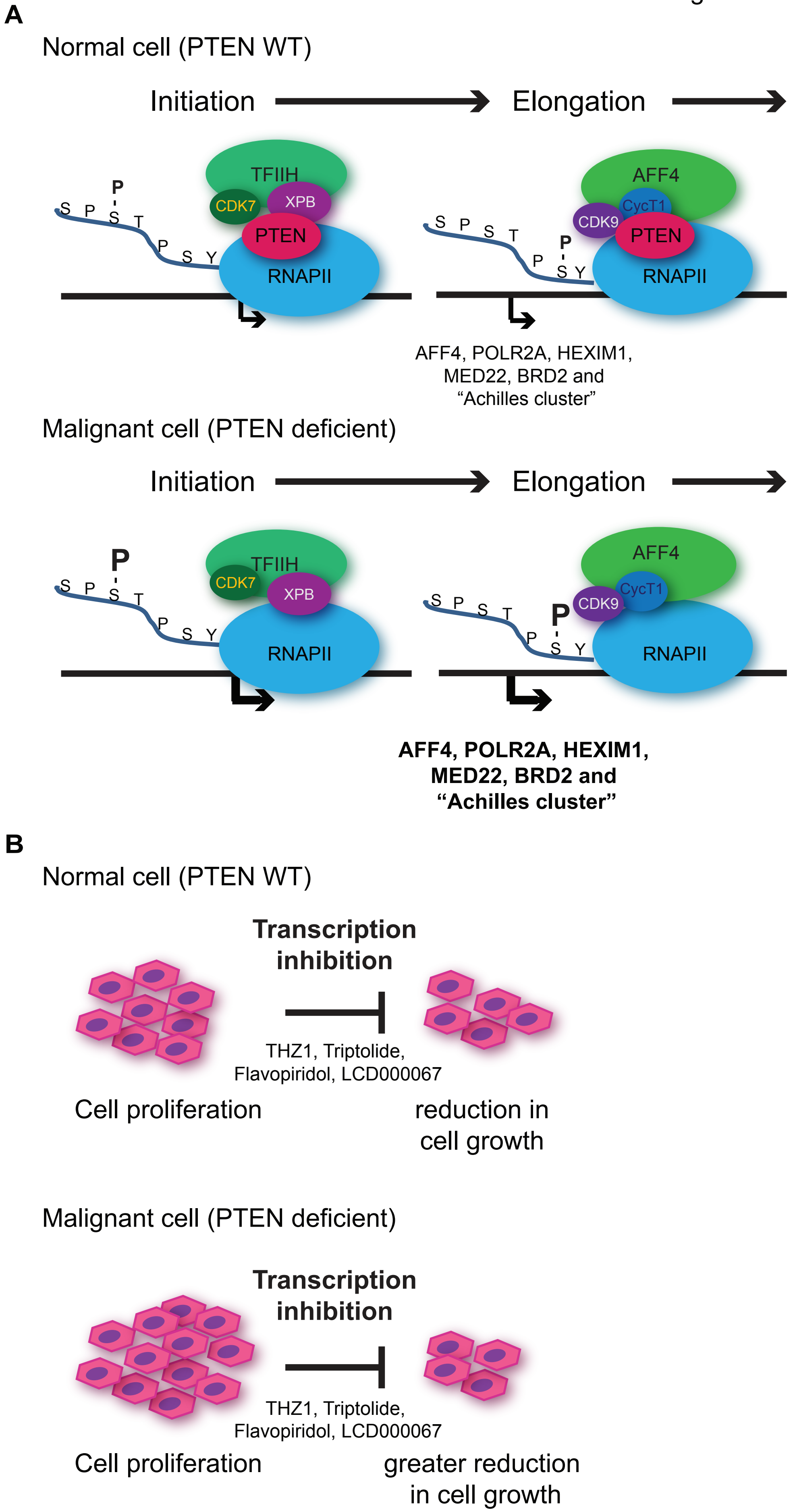
A
Normal cell (PTEN WT)
Initiation
Elongation
TFIIH
CDK7
XPB
PTEN
RNAPII
S P S T P S Y
P
AFF4
CycT1
CDK9
PTEN
RNAPII
AFF4, POLR2A, HEXIM1,
MED22, BRD2 and
“Achilles cluster”
Malignant cell (PTEN deficient)
Initiation
Elongation
TFIIH
CDK7
XPB
RNAPII
AFF4
CycT1
CDK9
RNAPII
AFF4, POLR2A, HEXIM1,
MED22, BRD2 and
“Achilles cluster”
B
Normal cell (PTEN WT)
Transcription
inhibition
THZ1, Triptolide,
Flavopiridol, LCD000067
Cell proliferation
reduction in
cell growth
Malignant cell (PTEN deficient)
Transcription
inhibition
THZ1, Triptolide,
Flavopiridol, LCD000067
Cell proliferation
greater reduction
in cell growth

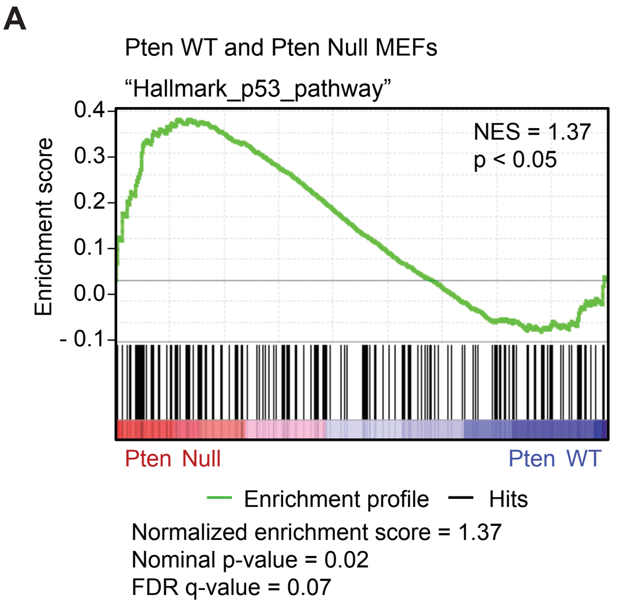
A
Pten WT and Pten Null MEFs
"Hallmark_p53_pathway"
NES = 1.37
p < 0.05
Enrichment score
0.4
0.3
0.2
0.1
0.0
- 0.1
Pten Null
Pten WT
Enrichment profile
Hits
Normalized enrichment score = 1.37
Nominal p-value = 0.02
FDR q-value = 0.07

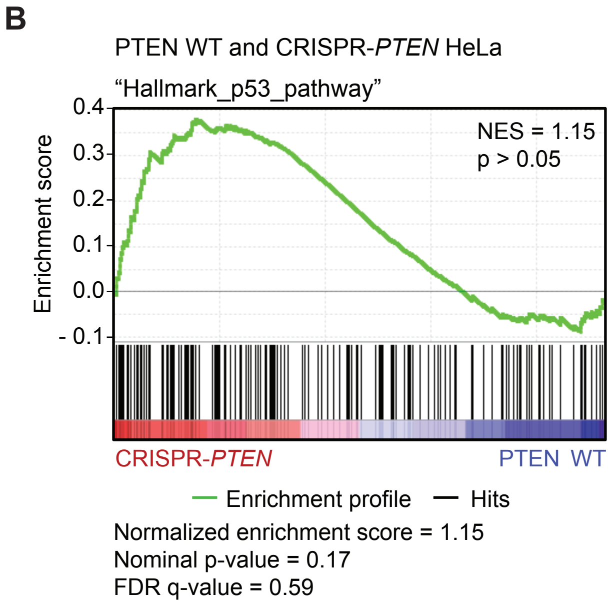
B
PTEN WT and CRISPR-PTEN HeLa
"Hallmark_p53_pathway"
NES = 1.15
p > 0.05
Enrichment score
0.4
0.3
0.2
0.1
0.0
- 0.1
CRISPR-PTEN
PTEN WT
Enrichment profile
Hits
Normalized enrichment score = 1.15
Nominal p-value = 0.17
FDR q-value = 0.59
